# A Gene Signature Derived from the Loss of CDKN1A (p21) Is Associated with CMS4 Colorectal Cancer

**DOI:** 10.3390/cancers14010136

**Published:** 2021-12-28

**Authors:** Santiago Bueno-Fortes, Julienne K. Muenzner, Alberto Berral-Gonzalez, Chuanpit Hampel, Pablo Lindner, Alexandra Berninger, Kerstin Huebner, Philipp Kunze, Tobias Bäuerle, Katharina Erlenbach-Wuensch, José Manuel Sánchez-Santos, Arndt Hartmann, Javier De Las Rivas, Regine Schneider-Stock

**Affiliations:** 1 Bioinformatics and Functional Genomics Group, Cancer Research Center (CiC-IMBCC, CSIC/USAL/IBSAL), Consejo Superior de Investigaciones Científicas (CSIC) and University of Salamanca (USAL), 37007 Salamanca, Spain; sjbuenofortes@usal.es (S.B.-F.); aberralgonzalez@usal.es (A.B.-G.); jose@usal.es (J.M.S.-S.); 2 Experimental Tumor Pathology, University Hospital of the Friedrich-Alexander University Erlangen-Nürnberg, 91054 Erlangen, Germany; jumuenzner@gmail.com (J.K.M.); chuanpit.hampel@uk-erlangen.de (C.H.); pablo.lindner@gmx.de (P.L.); alexandra.berninger@tum.de (A.B.); kerstin.huebner@uk-erlangen.de (K.H.); philipp.kunze@uk-erlangen.de (P.K.); 3 Institute of Pathology, University Hospital of the Friedrich-Alexander University Erlangen-Nürnberg, 91054 Erlangen, Germany; katharina.erlenbach-wuensch@uk-erlangen.de (K.E.-W.); arndt.hartmann@uk-erlangen.de (A.H.); 4 Preclinical Imaging Platform Erlangen (PIPE), Institute of Radiology, University Hospital Erlangen-Nuremberg, 91054 Erlangen, Germany; tobias.baeuerle@uk-erlangen.de; 5 Department of Statistics, University of Salamanca (USAL), 37008 Salamanca, Spain

**Keywords:** CDKN1A, consensus molecular subtypes (CMS), colorectal cancer, HCT116 cells, epithelial–mesenchymal transition (EMT), SNAI2, intermediate EMT, CAM model

## Abstract

**Simple Summary:**

A gene signature derived from the loss of *CDKN1A* (p21) gene, obtained in HCT116 p21-/- colorectal cancer cells, is identified in a large cohort of primary colorectal (CRC) tumors and is associated with the Consensus Molecular Subtype (CMS) of colon cancer that has a worse relapse-free and overall survival, that is, CMS4 (also called mesenchymal subtype). The presented gene signature can help to uncover the early molecular mechanisms of epithelial–mesenchymal transition (EMT), which is known to be associated with high stemness and drug resistance.

**Abstract:**

The epithelial–mesenchymal transition (EMT) is associated with tumor aggressiveness and increased invasion, migration, metastasis, angiogenesis, and drug resistance. Although the HCT116 p21-/- cell line is well known for its EMT-associated phenotype, with high Vimentin and low E-cadherin protein levels, the gene signature of this rather intermediate EMT-like cell line has not been determined so far. In this work, we present a robust molecular and bioinformatics analysis, to reveal the associated gene expression profile and its correlation with different types of colorectal cancer tumors. We compared the quantitative signature obtained with the NanoString platform with the expression profiles of colorectal cancer (CRC) Consensus Molecular Subtypes (CMS) as identified, and validated the results in a large independent cohort of human tumor samples. The expression signature derived from the p21-/- cells showed consistent and reliable numbers of upregulated and downregulated genes, as evaluated with two machine learning methods against the four CRC subtypes (i.e., CMS1, 2, 3, and 4). High concordance was found between the upregulated gene signature of HCT116 p21-/- cells and the signature of the CMS4 mesenchymal subtype. At the same time, the upregulated gene signature of the native HCT116 cells was similar to that of CMS1. Using a multivariate Cox regression model to analyze the survival data in the CRC tumor cohort, we selected genes that have a predictive risk power (with a significant *gene risk incidence* score). A set of genes of the mesenchymal signature was proven to be significantly associated with poor survival, specifically in the CMS4 CRC human cohort. We suggest that the gene signature of HCT116 p21-/- cells could be a suitable metric for mechanistic studies regarding the CMS4 signature and its functional consequences in CRC. Moreover, this model could help to discover the molecular mechanisms of intermediate EMT, which is known to be associated with extraordinarily high stemness and drug resistance.

## 1. Introduction

Colorectal cancer (CRC) represents one of the leading causes of cancer-related deaths worldwide, mainly due to its high metastatic rate. Approximately 20–30% of newly diagnosed CRC patients exhibit incurable and unresectable metastatic disease. Moreover, in patients with primary localized tumors, there is still a risk of recurrence and/or the formation of metastasis after resection of the primary tumor [1]. Metastasis formation is based on a multi-step process, that is also known as the invasion–metastasis cascade. It starts with the dissemination of cancer cells from the primary tumor site, followed by invasion into the surrounding tissue, intravasation, survival in the circulatory system, extravasation, and recolonization at a distant organ site; thus, eventually generating a secondary tumor [2]. All individual steps towards the formation of metastatic cells require specific changes and new features in the primary tumor cells, and these are largely connected to the epithelial–mesenchymal transition (EMT) and cancer stem cell phenotype [3]. Even though it is well known that the progression of the normal colon epithelium to an invasive and metastatic carcinoma is strongly associated with the process of EMT and the ability of tumor cells to survive under non-adherent conditions, the elucidation of the detailed mechanisms and regulators driving metastatic spread in patients remains a major focus for translational cancer research.

The cyclin-dependent kinase inhibitor p21 (i.e., the human gene *CDKN1A*) represents a negative regulator of both cell cycle progression and gene expression [4,5]. An uncontrolled passing of cells through the G1/S checkpoint by downregulation or loss of p21 might induce aberrant proliferation and, thus, trigger tumor transformation. In CRC, the downregulation of p21 expression has been reported to correlate with the development of metastases and poor patient survival [4,6]. In both, *Apc*+/- and *Muc2*-/- mouse models, the inactivation of p21 enhanced intestinal tumorigenesis [7,8]. It has also been shown that p21 knockout can induce EMT in non-tumorigenic mammary epithelial cells with long-term TGF-ß treatment [9,10]. These findings suggest that p21 might play a key role in inhibiting EMT, migration, and invasion. Indeed, Li et al. showed, for the first time, that p21 could form an inhibitory complex with the EMT transcription factor ZEB1 [5]. In this context, we recently reported that HCT116 p21-/- colon tumor cells exhibit a remarkable ZEB1-dependent deregulation of the epigenetic landscape [11]. Thus, p21-/- cells can be a suitable experimental model to study EMT-related processes in cancer, showing an intermediate phenotype between epithelial and mesenchymal characteristics, with only a partial loss of the adhesion marker E-cadherin [5].

In a pioneering work in 2015, Guinney et al. [12] proposed the existence of four consensus molecular subtypes (CMSs) of CRC, in addition to the classical histomorphological categories. So far, this classification system represents the most robust system available that can be applied for patient stratification and treatment strategy selection on the basis of specific biological and biomolecular characteristics [13]. According to Guinney et al., [12] the CMS4 subtype reflects the gene signature of mesenchymal cells, together with upregulated TGF-ß signaling and matrix remodeling. Interestingly, tumors of the CMS4 subtype were also majorly connected to drug resistance and increased tumor budding [14]. To our knowledge, a detailed gene signature profile of HCT116 p21-/- cells, in terms of CMS classification, has not yet been determined. Thus, we asked the question of whether a HCT116 p21-/- isogenic cell line showing an intermediate EMT with a gain in Vimentin levels, decreased E-Cadherin levels, and a reorganized epigenetic landscape might have changed the CMS subtype category of the microsatellite instable mutator HCT116 cell line. These cells were previously classified as CMS1 by Linnekamp et al. [15]. We generated a gene signature that was derived from the differential expression between the HCT116 p21-/- cells and the HCT116 cells and compared the signature with those proposed as characteristic signatures for CMS1 and CMS4 by Guinney et al. [12]. Then we verified our findings in a large independent cohort of human CRC samples with complete transcriptomic data. Finally, the prognostic value of the extracted gene signature for the CMS4 subtype was analyzed.

## 2. Results

### 2.1. p21 Loss Induced a Mesenchymal Phenotype in HCT116 Colorectal Cancer Cells

**Figure 1:**
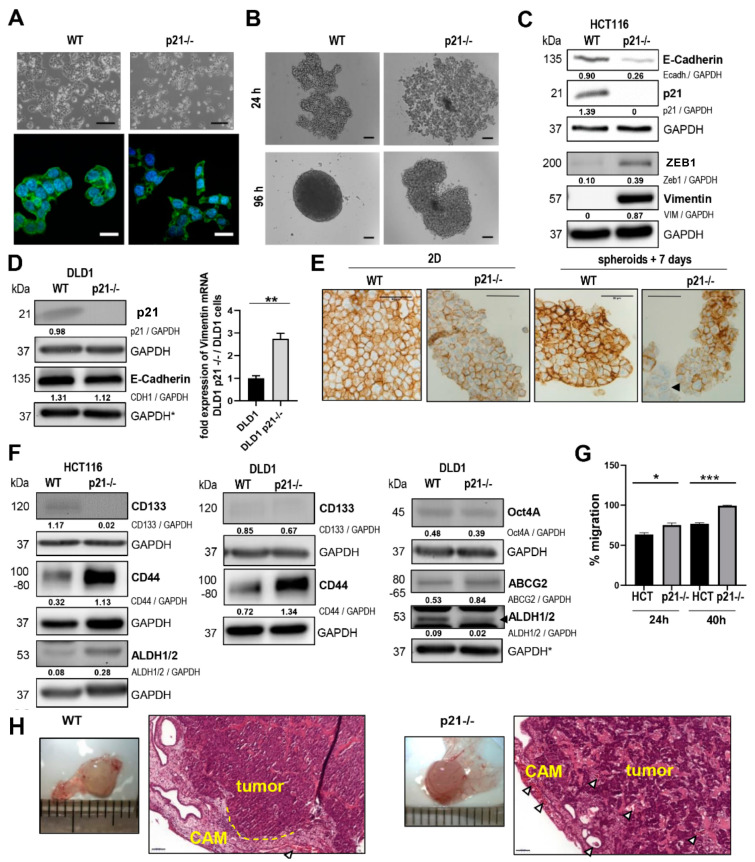
Comparison of colon cancer HCT116 WT versus HCT116 p21-/- cells. (**A**) Phase contrast and immunofluorescence confocal microscopy images of HCT116 WT and p21-/- cells. Top panels: phase contrast magnification = 100×; panels below: confocal magnification = 630×, blue: DAPI, green: F-Actin (scale bar of Phase contrast = 200 µm, confocal scale bar = 20 µm; *n* ≥ 4). (**B**) Spheroid formation ability of HCT116 WT and p21-/- cells in a hanging drop assay after 24 and 96 h (scale bar = 500 µm). (**C**) Western blots of HCT116 WT, HCT116 p21-/-, (**D**) DLD1 WT, and DLD1 p21-/- cells grown in 2D cultures, used to analyze the expression of EMT markers (Vimentin, ZEB1) and p21 and the epithelial marker E-cadherin (CDH1); *n* ≥ 2. Fold expressions are presented relative to GAPDH loading control. * Markers were blotted on the same gel. Fold changes of *VIM* expression in DLD1 p21-/- cells when compared to WT cells as determined by qPCR (** *p*-value < 0.01, *n* = 3; biological triplicate and technical triplicate). (**E**) Immunohistochemical staining for E-cadherin in fixed cells grown in 2D cultures and 3D cultures with hanging drops (scale bar = 50 µm). (**F**) Western blots of HCT116 WT, HCT116 p21-/-, DLD1 WT, and DLD1 p21-/- cells to investigate the stemness markers CD133, CD44, ALDH1/2, OCT4A, and ABCG2 (*n* ≥ 2). Fold expressions presented relative to GAPDH loading control. * Markers were blotted on the same gel (**G**) Evaluation of the wound healing assay after 24 and 40 h for cells treated with 10 nM of mitomycin C (average of 7 wells with 3 single measurements per well) (Image-Pro Plus). (* *p*-value < 0.05, *** *p*-value < 0.001). (**H**) Representative light microscopy images of fixed and extracted chorioallantoic membrane (CAM) tumors derived from HCT116 WT and p21-/- cells (magnification = 100×; scaling segments = 1 mm; *n* ≥ 8) and representative images of formalin-fixed and paraffin-embedded (FFPE) CAM sections stained with HE for histological analysis (magnification = 200×; scale bar = 50 µm, *n* ≥ 8). Arrows show blood vessels and the yellow dotted line marks the pushing invasion front.

In 2014, Li et al. [5] reported that p21 (CDKN1A) -/- cells showed an induction of EMT in different cell culture model systems, including the established CRC cell line HCT116. Thus, as one of the first steps of the present study, the mesenchymal features of the HCT116 p21-/- cancer cell line were investigated via phenotypical characterization (Figure 1). For this, we analyzed the HCT116 p21-/- cells in comparison to the parental cell line HCT116 wild-type (WT) via both light and fluorescence microscopy (Figure 1A). The microscopy images clearly showed a rounded epithelial morphology for the parental HCT116 cells, as well as growth in densely packed adherent colonies. In contrast, the HCT116 p21-/- cells exhibited a spindle-like cell morphology, as well as extended cell protrusions (Figure 1A) and growth in smaller spheroids, with rather loose intercellular contacts (Figure 1B). Western blot analysis verified complete p21 knockout in HCT116 cells and detected shifts in the expression levels of EMT-associated proteins (Figure 1C). More specifically, we observed downregulation of the epithelial marker E-cadherin (CDH1), while there was a massive upregulation of the expression of the mesenchymal marker Vimentin (VIM), as well as upregulation of the EMT-associated transcription factor ZEB1.

Correspondingly, analysis of another DLD1 p21-/- cell line also revealed slightly lower levels for E-Cadherin in Western blotting and an upregulation of Vimentin, but only detectable at the mRNA level (Figure 1D), whereas upregulation of the EMT transcription factor ZEB1 has already been confirmed in Lindner et al., 2019 [11]. Immunostaining showed remarkably reduced membranous E-cadherin signals in fixed cells from 2D cultures and 3D hanging drop spheroids (Figure 1E). Interestingly, there was a highly variable pattern for stem cell markers. HCT116 p21-/- cells were characterized by a loss of stemness marker CD133 and a gain in CD44 and ALDH1/2 (Figure 1F). The decrease in *CD44* levels on transcriptional level (NanoString) and the increase on protein level suggest posttranscriptional regulation mechanisms. The DLD1 p21-/- cells showed also slightly reduced CD133 levels, and increased CD44 levels, but in contrast to HCT116 p21-/- cells they had reduced ALDH1/2 levels. The ABCG2 levels were increased and Oct4 did not differ significantly from the DLD1 cells. ABCG2 and Oct4 were not detectable at all in HCT116 and HCT116 p21-/- cells. As expected for the mesenchymal phenotype, the HCT116 p21-/- cells exhibited a higher migratory potential (Figure 1G). In the in vivo CAM model, HCT116 p21-/- tumor masses were rather loosely packed, exhibiting a high infiltrative growth pattern at the invasion front of the CAM xenografts, while the HCT116 WT cells showed more solid tumor growth patterns, with broad pushing invasion fronts (Figure 1H). When examining the tumor cell dissemination, we could observe a tendency for p21-/- cells to show a higher radiation efficiency when applying an in vivo imaging system, although this was found without reaching significance (Supplementary Figure S1).

### 2.2. Differential Gene Expression Signature of HCT116 p21-/- Colorectal Cancer Cells

**Figure 2:**
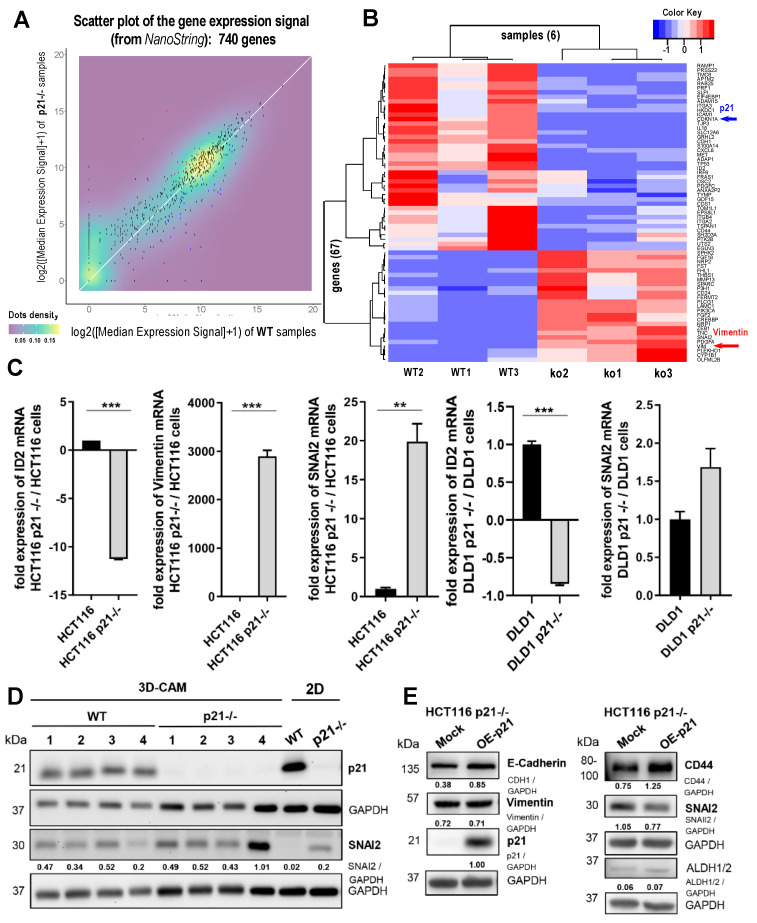
Differential expressions of colon cancer HCT116 WT versus p21-/- cells. (**A**) Scatter plot of the global expression signal. (**B**) Heatmap presenting the expression profiles of the 67 genes selected, in terms of the most significant differential expression changes found. Gene CDKN1A (*p21 protein) was not expressed in knockout samples, and gene VIM (vimentin) was a marker that was not expressed in WT samples. (**C**) Fold changes of expression ID2, VIM, and SNAI2 in HCT116 and DLD1 p21-/- cells when compared to WT cells, as determined by qPCR (** *p*-value < 0.01, *** *p*-value < 0.001, *n* = 3; biological triplicate and technical triplicate). (**D**) Western blot analysis of p21 and SNAI2 in CAM xenografts derived from HCT116 WT and HCT116 p21-/- cells (*n* = 4) and 2D culture (*n* = 2). Fold expressions are represented relative to GAPDH loading control. (**E**) Western blot analysis of p21 over-expression (OE) in HCT p21-/- cells to investigate EMT and stemness markers (*n* = 2). Fold expressions are presented relative to GAPDH loading control.

To gain a comprehensive view of the genes altered in HCT116 p21-/- colorectal cancer cells, we performed expression profiling of the knockout cells versus the parental cell line using the NanoString platform (as described in the Methods). The results of this analysis are presented in Figure 2 and in Table 1 and Supplementary Table S1.

In Figure 2A, a scatter plot of the gene expression signals obtained for the 740 measured genes is shown. The plot shows the comparison of the average expression signal (as median values) in the parental HCT116 WT cells versus the average signal in the HCT116 p21-/- cells. The statistical analysis of these data revealed a set of 155 genes showing the most significant and consistent changes (see Methods). Moreover, a subset of 67 genes within this list of differentially expressed genes (DEg) was also found in the list of differentially expressed genes of the CMS categories 1 and 4 (as described below in Table 1 and Supplementary Table S1). Table 1 summarizes the information for the 10 most upregulated genes and the 10 most downregulated genes found in the differential expression analysis, including a brief description of each gene. The expression profiles of the 67 genes found by comparison of three wild-type samples versus three knockout samples are illustrated in a heatmap in Figure 2B. The clustering of the samples was clear and the signature included 25 upregulated genes and 42 downregulated genes. Full details on the statistical parameters and significance of each of these 67 genes are included in Supplementary Table S1.

The data confirmed a significant downregulation of CDKN1A (p21) together with other expected repressed genes, such as E-cadherin (CDH1) and interleukin 18 (IL18). The data also revealed a significant overexpression of vimentin (VIM), together with SNAI2, FGF2, and ZEB1 (all upregulated genes were expected to increase with EMT). Therefore, the gene expression profiling confirmed the presence of key EMT protein markers for HCT116 p21-/- cells. VIM and ZEB1 upregulation were also confirmed by Western blotting from cells grown in 2D cultures (Figure 2C). qPCR analyses confirmed the downregulation of the DNA binding protein inhibitor ID2 and the upregulation of SNAI2 and Vimentin in HCT116 p21-/- and DLD1 p21-/- cells (Figure 2C). Moreover, the significant upregulation of the SNAI2 protein was verified in 3D CAM xenografts and 2D culture using Western blot analysis (Figure 2D). p21 overexpression in the HCT116 p21-/- cells resulted in a partial reversal of the phenotype, with a slight increase in E-Cadherin and decrease in SNAI2 levels but unchanged Vimentin levels in Western blotting (Figure 2E). Stem cell marker were not targeted, since ALDH1/2 did not change and CD44 levels were increased even further.

A more global analysis of the gene signature was performed by means of functional enrichment (Table 2), using the bioinformatics tool GeneTerm Linker (version available online in June 2019) [16]. Of the 25 upregulated genes, 23 were mapped in this database, as well as 40 of the 42 downregulated genes. This functional analysis indicated a clear upward regulation of genes associated with the cytoskeleton, extracellular matrix (ECM) composition, and cell adhesion, reflecting an enhanced interaction of the mesenchymal and highly migrating HCT116 p21-/- cells with their tumor microenvironment. Additionally, the gene signature was associated with alterations of biological processes related to cell adhesion and cytoskeletal components. In particular, the repressed signature included several genes involved in integrin signaling, such as ITGA2 (integrin α 2), ITGA3 (integrin α 3), and ITGB4 (integrin β 4), as well as the epithelial marker E-cadherin (CDH1).

Additionally, for a deeper exploration of the gene signatures found in our differential expression analysis, we compared the whole list of altered genes found with the NanoString PanCancer Progression Panel with the EMT signatures reported by Roepman et al. (2014), which contain 96 characteristic epithelial and mesenchymal markers [17]. In this analysis, we found a total overlap of 39 genes. Out of these, 22 genes were significantly deregulated in HCT116 p21-/- cells, when compared to the expression profiles of the parental HCT116 WT cell line. There were eight upregulated genes, namely, *SNAI2, SPARC, VIM, CD24, FBLN5, MGP, FN1,* and *VEGFB* (listed by decreasing fold-change); and 14 downregulated genes, namely, *ELF3, CDH1, CLDN7, CLDN4, MMRN2, CD44, TWIST1, MET, PRSS8, ITGB4, DSC2, SPINT1, KRT19*, and *SMAD3*. Most of these genes with altered expressions (i.e., 18 out of 22) perfectly matched the EMT expression profile described by Roepman et al. [17]. Moreover, nine of these genes were present in the selected list of 67 most significant genes (four upregulated: VIM, SPARC, SNAI2, CD24; and five downregulated: CDH1, CD44, MET, ITGB4, DSC2). We have marked these genes in a specific column in Supplementary Table S1, also indicating whether they corresponded to a mesenchymal or epithelial phenotype. The assignment to cell lines also revealed that most of the upregulated genes were mesenchymal markers, while the downregulated genes were epithelial markers.

### 2.3. Mapping the p21-/- Gene Signature on CMS Subtypes Using a Cohort of CRC Samples

**Figure 3:**
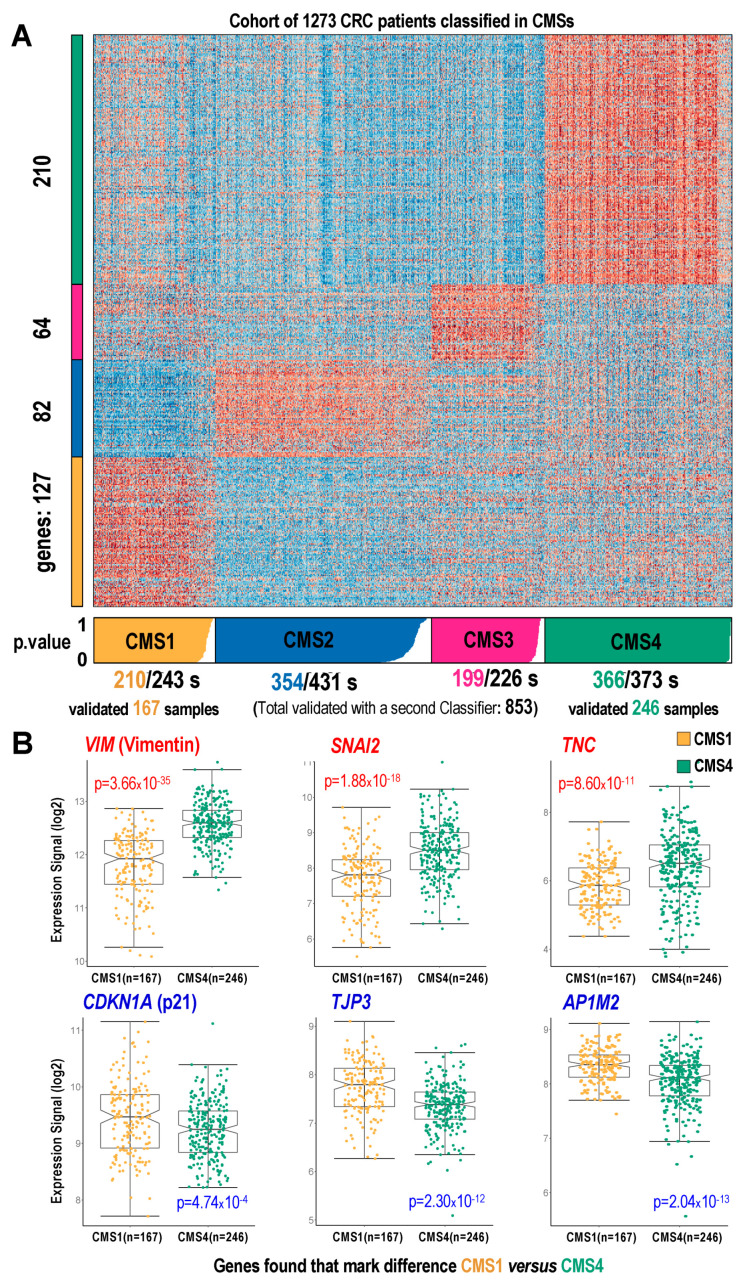
Comparative analysis of the expression profiles of a cohort of 1273 CRC patients, according to the consensus molecular subtypes (CMSs). (**A**) Heatmap presenting the expression; (**B**) expression signal comparison of 6 genes assigned to CMS1 samples versus CMS4 samples.

Next, we performed an independent validation using a cohort of 1273 tumor samples from patients with CRC. The cohort of CRC samples with normalized transcriptomic data and survival data was taken from our previous work [18]. Using this cohort, we performed molecular classification of the samples according to the gene signatures defined for each of the four consensus molecular subtypes (CMS1, 2, 3, 4) [12]. The gene signatures were identified by analyzing the expression profiles corresponding to the specific gene sets that characterized each of the four subtypes, i.e., 127 genes for CMS1, 82 genes for CMS2, 64 genes for CMS3, and 210 genes for CMS4 (Figure 3A). Each sample in the CRC cohort was assigned to one of the four CMS subtypes using the classification algorithm *CMScaller* [19] (Figure 3A), which allows the assignment of each individual to a given subtype with a significant value (where the relevant *p*-values from 0 to 1 are marked as colored bar boxes below the heatmap, Figure 3A). This assignment resulted in 88.7% of the tumor samples from the cohort exhibiting significant *p*-values <0.05. This means that 1129 samples were clearly classified in one of the CMSs, showing the following distribution: 210 tumor samples assigned to CMS1; 354 to CMS2; 199 to CMS3; and 366 to CMS4.

The classification of CRC samples into subtypes 1 and 4 was then validated using a second independent algorithm called *CMSclassifier* [12]. This second classification procedure achieved an assignment of 854 samples (67% of the total), including 167 samples assigned to CMS1 and 246 samples assigned to CMS4. These samples were validated by both classification algorithms. Thus, we achieved a robust identification of the human CRC samples that were considered to belong to CMS1 and CMS4. In addition, the stratification of the tumor samples into CMS1 and CMS4 allowed us to further investigate the genes characteristic for the molecular signature of HCT116 p21-/- cells (i.e., the signature of the 67 genes defined above, included in Supplementary Table S1, which can be considered to be involved in the switch between CMS1 and CMS4.

Figure 3B shows some of the most highly significant changes between CMS1 and CMS4 samples, presenting the top three upregulated genes (*VIM, SNAI2*, and *TNC*) and top downregulated genes (*CDKN1A, TJP3*, and *AP1M2*). The lower *CDKN1A* (*p21*) gene expression in CRC samples assigned to CMS4 provides a good verification and validation of our observations in HCT116 p21-/- cells. The complete results corresponding to all 67 genes of our refined signature were included in Supplementary Table S1 and showed a clear correlation of genes upregulated in HCT116 p21-/- cells and CRC subtype CMS4 (all 25 of the upregulated genes showed a higher expression in CMS4), while genes downregulated in the HCT116 p21-/- cells were associated to the CRC subtype CMS1 (40 out of 42 genes showed a higher expression in CMS1).

### 2.4. Analysis of Survival and Risk of CRC Samples Classified as CMS1 and CMS4

Some previous reports have suggested that the outcome regarding survival and overall prognosis in patients with CRC subtype 4 (CMS4) should be worse than the outcome of CRC patients with other subtypes (CMS1, 2, 3). Even though there are controversial data in the literature, this observation on differential survival was reported in the pioneering analyses performed by Guinney et al. [12], where they defined the CRC subtypes. Using the available survival data of our cohort of 1273 CRC samples [18], we performed a Kaplan–Meier analysis to investigate the effects of the CMSs on patient outcomes.

**Figure 4:**
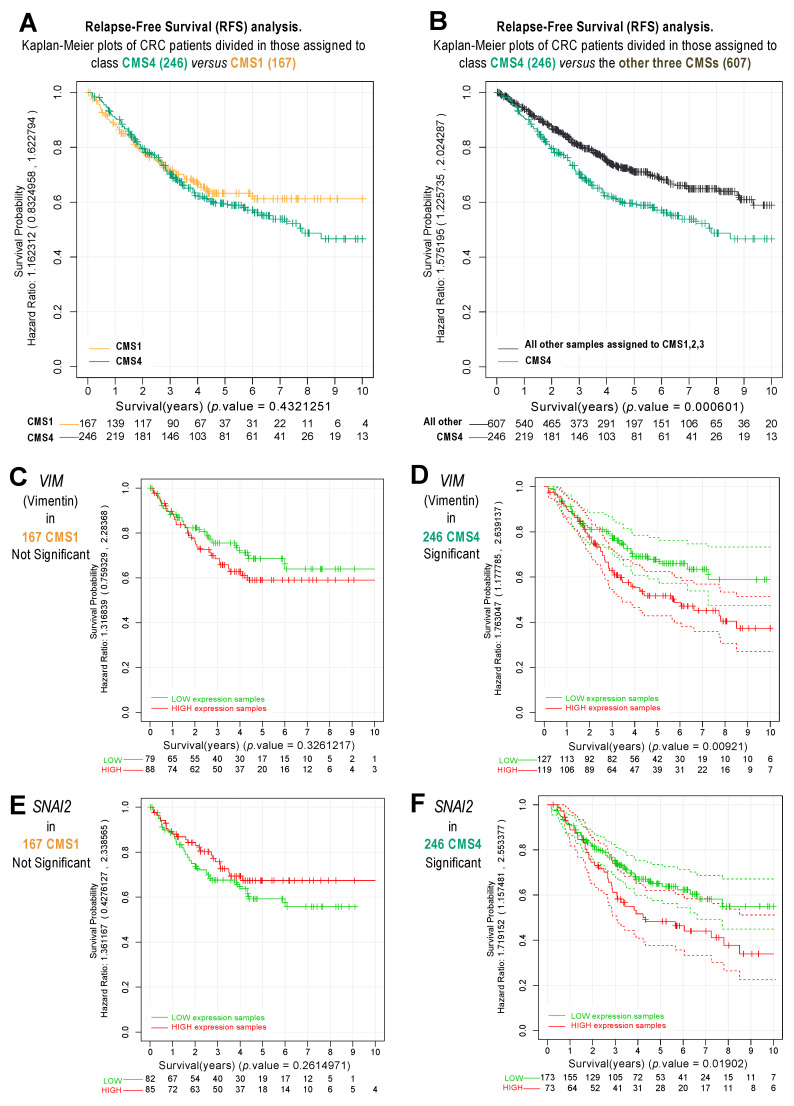
Kaplan–Meier survival analysis of CRC subtypes using the cohort of tumor samples presented in Figure 3. (**A**) Comparison of the relapse-free survival of the CRC samples identified as CMS4 (246) versus the CMS1 (167). (**B**) Comparison of the RFS of CMS4 (246) versus the ones assigned to all other CMSs (607 CMS1, 2, 3). (**C**) Comparison of the survival of 167 CMS1 samples, divided according to low or high expression of Vimentin. (**D**) Comparison of 246 CMS4 samples, divided by low or high expression of Vimentin. (**E**) Comparison of the survival of 167 CMS1 samples, divided according to high or low expression of SNAI2. **(F)** Comparison of 246 CMS4 samples, divided by high or low expression of SNAI2.

Figure 4 presents the Kaplan–Meier plots corresponding to the relapse-free survival (RFS) analysis of the CRC samples with different CMSs. First, following the arguments provided in the previous section, we performed a comparison of the survival of samples assigned to CMS1 and CMS4. This comparison did not show a significant difference between the survival of 167 CMS1 samples and the survival of 246 CMS4 samples (Figure 4A); however, we observed a significant difference between the survival of 246 CMS4 samples and the survival of all other samples assigned to subtypes 1, 2, and 3 (including 607 samples) (Figure 4B). This comparison presented a hazard ratio of 1.57 and a *p*-value of 0.0006. This result is in full agreement with the analysis reported by Guinney et al. [12], which showed poor prognosis for individuals with colon cancer subtype 4 (CMS4), when compared to all the other CMSs together (i.e., CMS1, 2, and 3). Thus, patients bearing a CMS4 tumor have a very high probability of developing a tumor relapse in a significantly shorter time than other CRC patients.

The Kaplan–Meier analysis of CRC patients presented in Figure 4A,B did not consider the gene expression values for the separation between different groups of patients, since the groups were classified according to their CMS assignment, as described in Figure 3A. However, the survival analysis can be performed using some specific genes to separate patients into two groups, using the median gene expression level as a cut-off value. This approach allows splitting the cohort in two groups, i.e., one with patients that present a high expression of a certain gene, and another with patients with a low expression of such a gene. We performed this separation using the two genes that were most significantly upregulated in HCT116 p21-/- cells (Table 1) and that were markers of the transition towards the mesenchymal state, namely *VIM* (Vimentin) and *SNAI2*. Furthermore, we conducted a study of the survival curves of these two genes, considering the two subsets of patients separately: CMS1 and CMS4. The results presented in Figure 4C–F indicate that the expression levels of *VIM* and *SNAI2* did not show significant differences within CMS1 samples (Figure 4C,E); although in the case of the CMS4 samples, the overexpression of *VIM* or *SNAI2* was significantly associated with shorter recurrence-free survival (RFS) (Figure 4D,F) and, thus, poor prognosis. 

These results confirmed the suggestion that genes upregulated in CMS4 correspond to a higher risk and a worse prognosis in this specific subtype of colorectal tumors. In addition, the results led to the conclusion that the CMS-specific gene signatures clearly determine a specific CMS biological behavior. When analyzing p21, *p21* expression levels did not allow for a significant segregation between CRC patients regarding their survival. We analyzed two different expression datasets, namely (i) a gene expression dataset with 545 CRC tumor samples, where the different *p21* levels were not significantly correlated with survival (*p*-value = 0.51); and (ii) another protein expression dataset from the Human Protein Atlas with 597 CRC tumor samples (*p*-value = 0.16). Thus, these additional results suggest that p21, by itself, is not a suitable prognosis marker (neither at the mRNA level nor at the protein level).

We might speculate that low p21 expression is necessary but insufficient to determine poor prognosis, and that additional factors are involved. To clarify this point we performed a survival analysis in our cohort of patients, using the 67-gene signature that we had identified.

As noted above, to clarify whether a lack of p21 was associated with a poor prognosis, and whether additional factors were involved, we performed a survival analysis in our cohort of patients using the gene signature we had identified (which included 67 genes).

**Figure 5:**
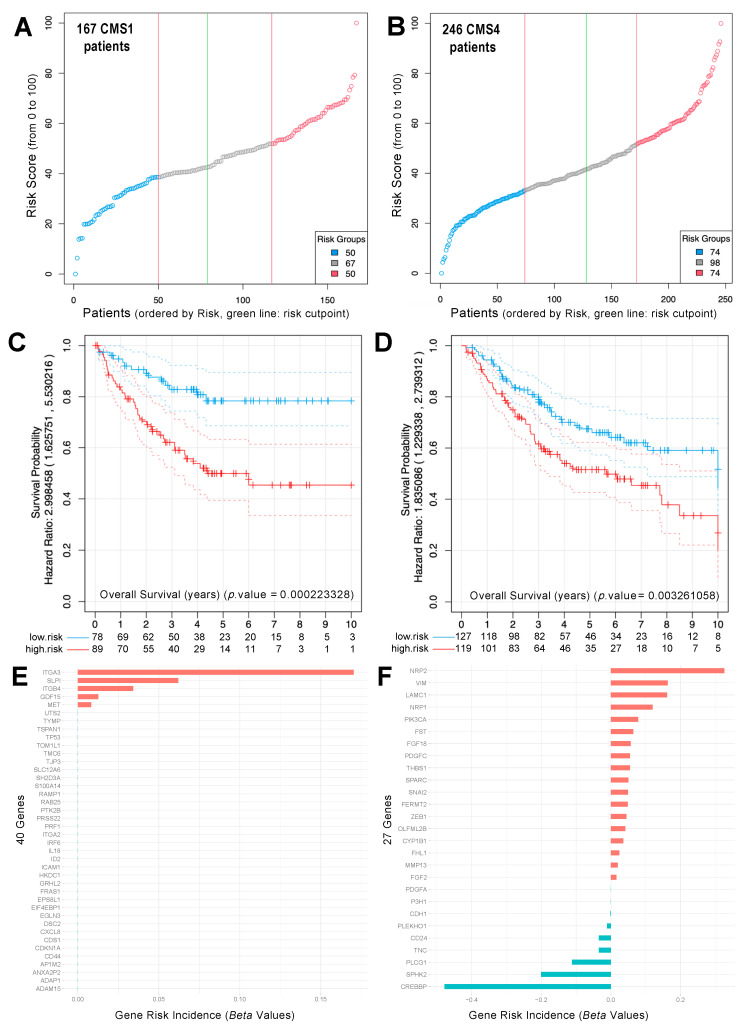
Risk prediction of CRC tumor samples using the gene signature of 67 genes (40+27) identified in p21 depleted cells. A relative value-of-risk score (from 1 to 100%) was assigned to each patient included (**A**) in the CRC tumor samples classified as CMS1 (167 samples) using the signature of 40 genes that are upregulated in the cells with p21; or (**B**) in the CRC tumor samples classified as CMS4 (246 samples), using the 27 genes that are upregulated in the cells that do not have p21. Once each patient’s risk was calculated, a Kaplan–Meier analysis tested the separation of the two groups according to the survival: a high-risk group of individuals with poor survival (in red), and a low-risk group of individuals with good survival plotted (in blue). The *p*-value of the log-rank test, to evaluate the difference between the Kaplan–Meier curves of these two groups of patients for each gene signature, is presented in plots (**C**,**D**). Finally, two plots with the gene risk incidence scores (i.e., the beta values) of the genes tested in the risk prediction of the CMS1 samples (**E**) and the CMS4 samples (**F**) are presented.

**Figure 6:**
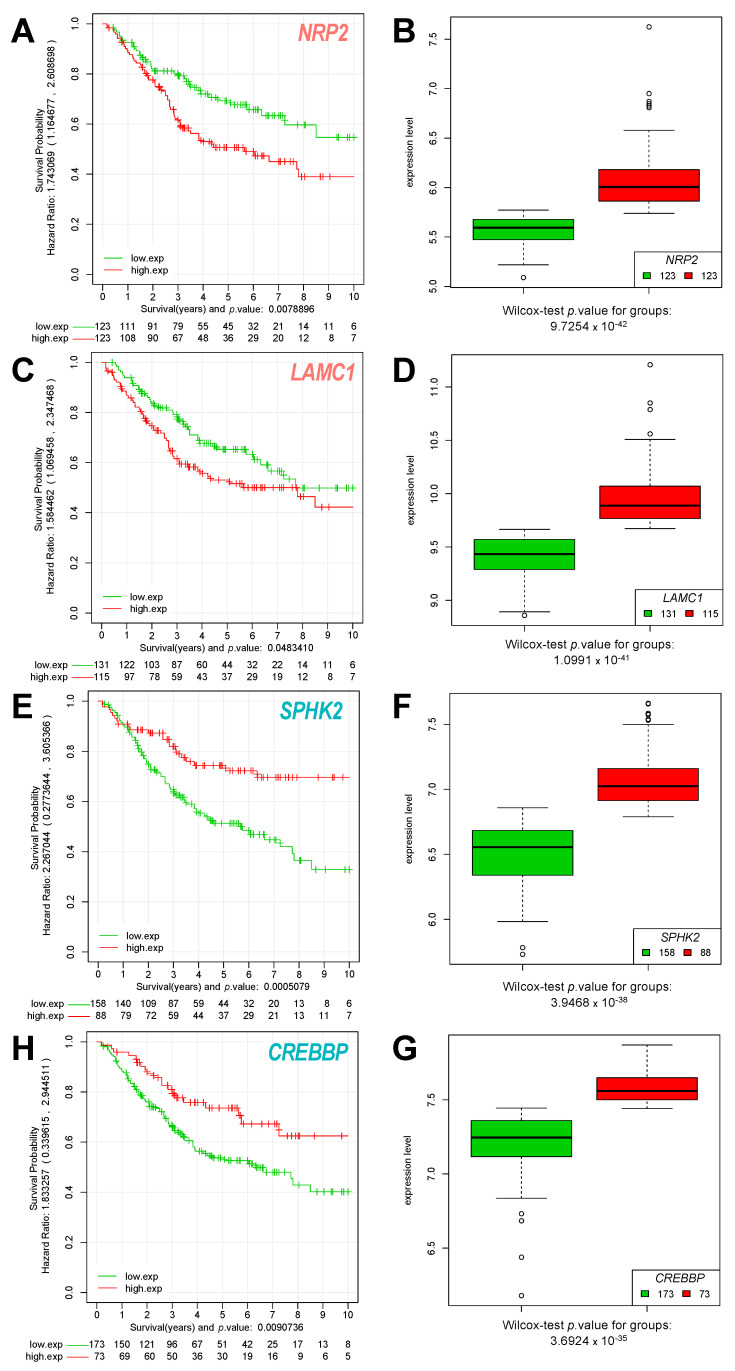
Kaplan–Meier survival analysis of individual genes identified by risk prediction of CRC tumor samples of CMS4 subtype (246 samples). The genes presented are NRP2: (**A**) Kaplan–Meier (KM) curve corresponding to the samples divided by (**B**) the expression level of the gene (high expression red, low expression green). Gene LAMC1: (**C**) KM curve of the samples divided by (**D**) the expression level. Gene SPHK2: (**E**) KM curve of the samples divided by (**F**) the expression level. Gene CREBBP: (**H**) KM curve of the samples divided by (**G**) the expression level.

The analysis revealed a clear separation of CRC primary tumor samples, regarding survival when performing the risk prediction in the set of 167 CMS1 patients with the 40 upregulated genes in HCT116 p21-/- cells from our gene signature (Figure 5A–D). Thus, we suggest that these genes clearly contribute to the risk of CMS1 patients, with ITGA3/ITGB4 being among the most prominent candidates (Figure 5E, Supplementary Figure S2). At the same time, we also showed a strong separation of tumors with poor and good survival when performing the CRC patient risk prediction in the set of 246 CMS4 patients with the 27 genes upregulated in our proposed gene-signature in the HCT116 p21-/- cells (Figure 5D). We suggest that these genes are only relevant risk factors in CMS4 patients, allowing a clear risk discrimination for this class of patients. Here, besides *SNAI2* and *ZEB*1, two well-known EMT regulators, new interesting candidates also appear, such as cytoskeleton-associated NRP1/2 or ECM interacting protein LAMC1, associated with worse prognosis, and SPHK2 and CREBBP, associated with good prognosis (Figure 5F and Figure 6A–G). *ITGB4*, as upregulated in the CMS1 CRC subtype, was associated with poor survival only in the CMS1 group without, or with only marginal, predictive value in CMS4 tumors (Supplementary Figure S2).

### 2.5. Functional Differences in Genes Included in CRC Subtypes CMS1 and CMS4

In order to stratify the characteristics of the tumor samples assigned to CMS1 and CMS4 in our CRC cohort, we performed a functional enrichment analysis, to identify the biological processes that were overrepresented in these subsets of CRC samples. We expected to identify functions attributed to CMS1 and CMS4. 

**Figure 7:**
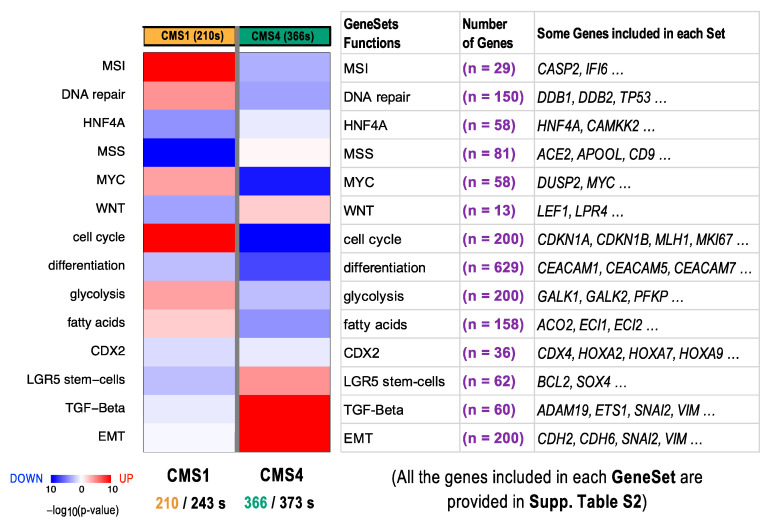
Functional enrichment analysis of the genes included in the CRC subtypes CMS1 and CMS4 based on the gene expression profile of the patients assigned to these two subtypes. All the genes included in each functional gene set are provided in Supplementary Table S2. MSI: microsatellite instability; MSS: microsatellite stability.

To do this, we selected the samples that were classified in CMS1 and CMS4 via *CMScaller* [19]. In this way, a whole set of 576 samples (210 CMS1 and 366 CMS4) was used, and the transcriptomic expression profile was tested for functional enrichment versus the 14 gene sets provided by Eide et al. [19] (Figure 7). These gene sets define biological functions relevant in cancer, tumor progression, and metastasis. Nine of them were derived from the Molecular Signatures Database (MSigDB, http://software.broadinstitute.org/gsea/msigdb/, last accessed 10 October 2021), including the CDX2 set (with 36 genes), cell cycle set (200 genes), DNA repair set (150 genes), EMT set (200 genes), glycolysis set (200 genes), HNF4A set (58 genes), microsatellite instability (MSI) set (29 genes), microsatellite stability (MSS) set (81 genes), and MYC signaling set (58 genes). The five other gene sets included in our analysis were the fatty acid metabolism set (with 158 genes) [20], gastrointestinal differentiation markers (629 genes) [21], LGR5 stem cell set (62 genes) [22], TGF beta signaling set (60 genes) [23], and WNT signaling set (13 genes) [24]. The heatmap presented in Figure 7 shows a significant upregulation of the CMS1 samples for the functional sets corresponding to the MSI and cell cycle versus a significant upregulation of TGF beta signaling and EMT in the case of the CMS4 samples. All genes included in each of the functional sets are provided in Supplementary Table S2.

## 3. Discussion

In this work, we have described the novel finding that the gene expression signature obtained with HCT116 p21-/- cells closely resembles the molecular subtype CMS4 of colorectal cancer in vitro and in vivo. We performed a differential expression analysis with human colorectal HCT116 p21-/- carcinoma cells versus HCT116 wild-type (p21 WT) cells using *NanoStringDiff* [25]; that is a robust statistical method applied to NanoString nCounter expression data. The algorithm is based on linear regression and has provided an accurate quantification of gene expression [25], producing a significant signature that includes multiple genes associated with the process of epithelial to mesenchymal transition (EMT) in colorectal cancer and metastasis [26,27]. Moreover, the high prognostic value of this signature has been verified in a CMS subtype specific manner.

In 2015, Guinney et al. [12] presented a novel method for the classification of CRC patients, which did not only consider classical histomorphological features, but was largely based on gene expression profiles. Using a multi-classifier system, they defined four CMSs for CRC patients. Within this classification, CMS1 represents the so-called MSI hypermutated immune phenotype, characterized by an increased expression of genes linked to a strong infiltration of colorectal tumors with immune cells and the evasion of immune response (i.e., MSI immune subtype). CRC tumors of the CMS2 class displayed upregulation of WNT and MYC signaling and showed a distinct epithelial differentiation (also being considered tumors of the most classic canonical CRC type, i.e., the canonical subtype). CMS3 tumors were associated with a high deregulation of genes responsible for metabolic adaptation of cancer cells (i.e., metabolic subtype). Finally, tumors of CRC patients classified as CMS4 showed high expressions of EMT genes and gene expression profiles that could be connected to an activation of TGF-β signaling, tumor cell–matrix interaction, angiogenesis, and metastasis formation, as well as inflammation (i.e., mesenchymal subtype). CMS4 tumors with these mesenchymal features were found to be the most aggressive, and patients harboring these tumors showed poorer overall survival and relapse-free survival [12]. Furthermore, in a classification of colorectal adenomas (i.e., early colorectal tumors), the CMS4 subtype was not detected, as there were no invasion-associated stromal phenotypes in these early-stage tumors [28].

The accurate classification of tumors might be difficult for preclinical models since patient-derived xenografts (PDX) or xenografts from established tumor cell lines have been shown to adapt to the metabolic profile of the host mouse over time [29]. Despite this, Linnekamp et al., in 2018 [15], demonstrated that several CRC cell lines could be classified successfully using an adapted CMS classification system. In fact, despite the established fact that cell lines lack interaction with other cell types or stromal components, a subset of the investigated CRC cell lines was clearly classified as CMS4, which is largely associated with stromal interaction. Furthermore, in this work, we bypassed the ‘stromal problem’ by using a bioinformatics workflow that applied a CMS classifier independent of stromal gene expression, suggesting that the gene signature of CMS4 clearly represents an intrinsic feature of the tumor.

In this study, we also used a large integrated CRC cohort to extract the gene signature of mesenchymal HCT116 p21-/- cells. This combined cohort corresponded to a curated normalized dataset that included 1273 CRC samples with genome-wide expression profiles plus disease survival information. The cohort was analyzed to identify the CMS1-4 molecular subtypes provided by Guinney et al. [12]. The results of our study added important knowledge about the suitability of HCT116 p21-/- cells as a model for mechanistic studies of the mesenchymal CMS4 subtype of CRC and its functional relevance. Although Linnekamp et al. [15] previously classified six colorectal tumor cell lines as representing CMS4, the direct comparison of the two isogenic HCT116 cell lines allowed a more refined study of the CMS1 ‘MSI immune’ subtype and CMS4 ‘mesenchymal’ subtype within a very similar and homogeneous genetic background. When considering the 740 Nanostring gene panel, we found differences in 67 genes, which represented less than 10% of the entire panel, but which had the power to switch the molecular subtype of the cells.

Our results also identified several novel genes that have so far not been associated with the standard processes related to EMT. As an example, the role of ID2 in EMT for colorectal cancer has not been previously described. It has been reported that the upregulation of ID2 in breast cancer is associated with a reduced vimentin expression and attenuated EMT features in triple-negative breast carcinomas [30]. In contrast, in a hepatocellular carcinoma, ID2 upregulation triggered invasion and metastasis via EMT induction. Obviously, the functional role of ID2 can be very context-specific and must be carefully evaluated in terms of CRC. In any case, the comparison using two HCT116 cell lines, applied in our study as a model system, should at least minimize the genetic background dependency [31].

Even with being clearly classified as the CMS4 subtype, HCT116 p21-/- cells have not completely lost E-cadherin (CDH1) protein expression. As reported in a recent paper concerning tumor buds, single cells and small cell clusters (<5 cells) at the tumor invasion front of CRC show Vimentin/pan-Cytokeratin positive staining and are suggested to be in an intermediate state, with partial EMT. The existence of these double-positive buds in an atypical cancer-associated stroma was correlated with a highly aggressive tumor behavior [32]. In this regard, we showed loosely packed and infiltrative tumor masses of p21-/- cells in the in vivo CAM model, which is in contrast to a clear pushing front, as can be normally observed for microsatellite instable tumors, such as those formed by HCT116 WT cells. Thus, HCT116 p21-/- cells could help to discover the molecular mechanisms of partial or hybrid EMT, which is known to be associated with extraordinary high stemness and drug resistance [33]. Indeed, the upregulated stemness marker ALDH1/2 in p21-/- cells, which is known to detoxify chemotherapeutic agents, contributes to drug resistance. We also detected higher CD44 levels. Circulating double positive CD44 and ALDH1/2 cells were shown to have an increased capacity for tumor initiation and are characteristic for cancer stem-like cells [34]. As found in the risk prediction analysis of our identified 67-gene signature, overexpressed Neuropilin (NRP2) and LAMC1 also appeared as determinants of worse prognosis. NRP2 is known to trigger EMT, migration, and metastasis in epithelial cancers [35]. LAMC1, as a member of the laminin family, plays an important role in ECM interaction but its role in EMT is not well-understood. The role of the overexpressing epigenetic modulators SPHK2, CREBBP, and others as predictors of good prognosis in patients with CMS4 tumors needs to be further elucidated.

Pathway enrichment analysis of the serrated alternative pathway of colorectal carcinogenesis has revealed that sessile serrated adenomas represent both CMS1-like and CMS4-like features [36]. From our transcriptional profiling and bioinformatics analysis, we could speculate that the CMS1 and CMS4 subtypes are closer to each other than to CMS2 or CMS3. Indeed, CMS1 and CMS4 showed the lowest expressions of genes associated with colonic epithelial differentiation [12]. Since CMS1 is hypermethylated, we could also speculate that the loss of p21 induced some kind of genome demethylation and, consequently, a novel gene expression signature; however, when measuring the 5-mC levels of long interspersed nucleotide element 1 (LINE-1) repeats, which serve as surrogate markers for global DNA methylation levels, we did not detect large differences between the HCT116 and HCT116 p21-/- cells (data not shown). Nevertheless, we cannot neglect the remarkable deregulation of epigenetic markers, such as histone modifying enzymes, in HCT116 p21-/- cells (as described by Lindner et al., in 2020) [11], which might have an important influence on the generation of the new gene expression signatures. The power of the loss of p21 to switch molecular subtypes from CMS1 to CMS4 is quite impressive, since p21 itself is not a transcription factor. We did not expect p21 knockout to cause so many alterations and can speculate that the loss of the p21 scaffold function might be responsible for the dramatic change in the CMS subtype gene signature [10]. Indeed, p21 has a plethora of different interaction partners [4], which could explain why p21 loss induces such a multifaceted phenomenon, as EMT, which involves proliferation, migration, invasion, angiogenesis, and differentiation processes. Although there was a tendency to rescue the epithelial phenotype when p21 was overexpressed, we do not believe that a cell line passaged and cultivated for more than 25 years [37] can be fully rescued with a simple p21 transfection. The cell line has completely adapted to the loss of the p21 gene and might have irreversibly changed its gene expression pattern. Regarding Gartel, who proposed in 2009 the idea of ‘antagonistic duality’ for p21 in cancer, acting as an oncogene or as a tumor suppressor gene in a context-dependent manner, this paradox might explain why p21 is not qualified as a prognostic marker in CRC [38]. Nevertheless, the HCT116 p21-/- cell line is a versatile tool for the mechanistic study of functional readouts of the CMS4 gene signature.

Taken together, our results prove the classification of the HCT116 p21-/- cell line as being of the CMS4 subtype of CRC. This finding offers the exceptional possibility of studying the set of genes responsible for this classification and CMS switching, not only in this individual cell line, but also in comparison to the isogenic CMS1 HCT116 WT cell line; thus, providing model systems with highly compatible genetic backgrounds to unravel novel EMT/partial EMT-dependent functions in cancer. Furthermore, our comprehensive analysis on CRC tumor samples gives preliminary hints that the switch from CMS1 to CMS4 might be independent of the microsatellite instability, hypermethylation, or mutation status of the cancer driver BRAF.

## 4. Material and Methods

### 4.1. Human Colorectal Carcinoma Cell Lines

HCT116, isogenic HCT116 p21-/-, DLD1, and isogenic DLD1 p21-/- cell lines were used for this study. HCT116 cells were obtained from the American Type Culture Collection (ATCC, Manassas, VA, USA), while the HCT116 p21-/- cells were a kind gift from Bert Vogelstein (Johns Hopkins University School of Medicine, Baltimore, MD, USA). DLD1 and DLD1 p21-/- cells were purchased from Horizon Discovery Group plc. HCT116 p21-/- cells were cultured in Dulbeco’s modified Eagle’s medium (DMEM; (Life Technologies, Darmstadt, Germany) supplemented with 10% fetal bovine serum (FBS, PAN Biotech, Aidenbach, Germany), 1% penicillin/streptomycin (PAN Biotech), 1% sodium pyruvate solution (Sigma-Aldrich, St. Louis, MO, USA), and 1% MEM Non-Essential Amino Acids solution (Life Technologies); while, HCT116, DLD1, and DLD1 p21-/- cells were cultured in RPMI supplemented with 10% fetal bovine serum (FBS, PAN Biotech) and 1% penicillin/streptomycin (PAN Biotech), at 37 °C in a humidified atmosphere with 5% CO_2_. The mycoplasma-free statuses of the cell lines were confirmed and their genotypes were authenticated using multiplex cell authentication by Multiplexion (Heidelberg, Germany).

### 4.2. Morphology and Fluorescence Staining

The HCT116 and HCT116 p21-/- cells were seeded on glass cover slips, allowed to attach, grown for 24 h, and then fixed in 4% phosphate-buffered formalin for 20 min at room temperature (RT). After washing, the permeabilization of cells was achieved with 0.2% Triton X-100 in phosphate-buffered saline (PBS) (Sigma) for 5–10 min at RT. Cells were then washed again with PBS and incubated with a blocking buffer (1% BSA in PBS) for 10 min at RT. Finally, another washing step (PBS) followed by fluorescence staining of F-actin was performed with Alexa Fluor^®^ 488 fluorescent dye (Life Technologies) for 30 min at RT, and cells were mounted on microscopy slides using the ProLong^®^ Gold Antifade reagent with DAPI (Life Technologies). Confocal (fluorescence) images were acquired using laser scanning microscopy (LSMT-PMT Observer Z1, Carl Zeiss, Oberkochen, Germany) and the ZEN imaging software (Carl Zeiss) with a 63× oil objective. Images were edited using the ZEN imaging software, Adobe PhotoShop (version CS5), and ImageJ (version 1.52a, NIH, Madison, WI, USA).

### 4.3. Collection of Cell Pellets for Western Blot, RT-qPCR, and NanoString Analysis

Tumor cells were harvested at 80% confluence. For this, each cell culture plate was placed on ice and cells were scraped into cell culture media. The cell suspensions were then transferred into 50 mL tubes. After washing the cell culture plate with ice-cold PBS to recover all remaining cells and transferring them into respective tubes, cells were centrifuged for 5 min at 5000 rpm and 4 °C. The supernatant was discarded, the cell pellet was resuspended in ice-cold PBS, and the cell suspensions were transferred into 1.5 mL tubes. Following another centrifugation step with the same conditions, cell pellets were frozen in liquid nitrogen and stored at −80 °C until further use. This whole process was repeated to obtain three independent biological replicates of the HCT116 WT cells and three replicates of the HCT116 p21-/- cells.

### 4.4. Western Blotting

The cell lysis, determination of protein concentrations, protein separation by SDS-PAGE, and blotting procedures were performed as previously described [39,40]. For the detection of specific protein bands, the following antibodies were used: p21 Waf1/Cip1 (12D1, Cell Signaling #2947, 1:2000), ZEB1 (D80D3, Cell Signaling #3396, 1:500), Vimentin (D21H3, Cell Signaling #5741, 1:5000), E-cadherin (24E10, Cell Signaling #3195, 1:2000), SNAI2 (Slug, C19G7, Cell Signaling #9585, 1:1000), CD133 (Miltenyi #130-092-395, 1:250), ALDH1/2 (Santa Cruz #sc-166362, 1:500), CD44 (Cell Signaling #3570, 1:1000), ABCG2 (Cell Signaling # 4477, 1:1000), Oct4A (C52G3, Cell Signaling #2890, 1:1000), and GAPDH (6C5, Abnova #MAB5476, 1:40,000). Protein bands were visualized using Immobilon Western Blot Chemiluminescent HRP Substrate (Merck Millipore, Burlington, MA, USA). Images were processed and analyzed using ImageJ (ImageJ 1.46r, Rasband, WS, U.S. National Institutes of Health) and ratios were determined relative to the intensity of the housekeeper GAPDH band. The uncropped western blot figures can been found in supplementary files.

### 4.5. 3D Spheroid Generation and Immunostaining

In total, 20 µL of cell suspensions (3 × 10^3^ cells each) was pipetted into the lid of a 10 cm culture dish as individual droplets. The lid was then inverted and put back onto the dish. Spheroids were harvested after 7 days of incubation at 37 °C, rinsed with PBS, and then gently centrifuged (700 rpm for 5 min). For the immunostaining of spheroids, they were fixed in 4% phosphate-buffered formalin for 24 h and embedded in paraffin. The 3-µm-thick spheroid slices were stained with an antibody against E-cadherin (dilution 1:2000, BD Bioscience, Franklin Lakes, NJ, USA) and counterstained with hematoxylin (Merck KGaA, Darmstadt, Germany). To monitor spheroid growth microscopically over time, spheroids were transferred to 6-well plates and analyzed daily.

### 4.6. Wound Healing Assay

A cell suspension at a density of 3 × 10^5^ cells/mL (70 μL volume) was applied to each chamber of a cell culture insert (Ibidi, Munich, Germany). The cell culture insert was removed after the cells had attached to the plate overnight. A new medium containing 10 nM mitomycin C (cell proliferation inhibitor) was added. Images were captured at 0, 24, and 40 h of incubation using a phase contrast microscope. The cell-free areas were measured with the Image-Pro Plus software (version 1.52a, Media Cybernetics, Rockville, MD, USA) and relative cell migration was quantified by the following equation: %Migration = [1 − (cell-free area at *t*_24_/cell-free area at *t*_0_) × 100].

### 4.7. Gene Expression Measurement with the NanoString Platform

Gene expression measurements were performed using the PanCancer Progression Panel (a multiplex transcriptomic platform) from NanoString (https://www.nanostring.com/, last accessed 15 June 2021), which included 770 human genes related to cancer progression. The gene expression assays were produced according to the manufacturer instructions, i.e., using 100 ng of total RNA for each sample as input. Three biological replicates were produced and tested for both the HCT116 p21-/- samples and the HCT116 WT control samples. Raw data (counts per analyzed gene) were obtained using the nCounter^®^ MAX/FLEX Analysis System following the method provided by the manufacturer. An independent analysis of the raw counts of the samples was done using R and the method provided by the package, called NanoStringDiff from Bioconductor (https://bioconductor.org/, last accessed 10 June 2021), as developed for differential expression analysis of NanoString nCounter data [25]. This algorithm is based on linear regression and uses a generalized linear model (GLM) of the negative binomial family to characterize count data; thus, allowing for multivariate analysis (i.e., multifactor design). Data normalization was incorporated into the model framework through three normalization parameters, namely, (i) positive controls, (ii) negative controls, and (iii) housekeeping genes, as provided by NanoString nCounter. In this way, the NanoStringDiff algorithm incorporates the model size factors calculated from the positive controls and the housekeeping controls included in the panel, as well as the background level estimation obtained from the negative controls [20]. The method also included an empirical Bayes shrinkage approach to estimate the dispersion parameter in the model and a likelihood ratio test to identify differentially expressed genes in a robust way. The use of this method provided a robust calculation of the normalized expression signal of each gene in each sample. The final number of measured human genes was 740, since the NanoString PanCancer platform incorporates 30 housekeeping genes. The platform also includes 6 positive controls and 8 negative controls, which, as indicated above, were used for the background correction and intra-sample and inter-sample normalization.

### 4.8. Differential Expression Calculation and CMS Gene Signature Identification in CRC Cohort

Differential expression analysis between the 3 HCT116 p21-/- samples and 3 HCT116 WT controls was performed using the method provided by the *NanoStringDiff,* based on linear regression [25]. This analysis provided a list of 378 differentially expressed genes with an adjusted *p*-value <0.05; and a more significant sub-list of 155 genes with an adjusted *p*-value < 1.0 × 10^−9^. This gene set was further evaluated using a cohort of 1273 CRC samples from patients [18], which had been classified according to the subtypes (CMSs) defined by Guinney et al. [12]. The classification of these tumor samples was performed using two algorithms developed for this purpose: *CMScaller* [19] and *CMSclassifier* [12]. The genes that presented significant differences between CMS1 and CMS4 in this large cohort of human tumors were selected and compared with the set of 155 genes identified in the analysis of HCT116 cells described above. In this way, we obtained a signature of 67 genes that characterize the p21-/- condition in the cell lines, and these were assigned to CMS1 or CMS4 according to their expression profiles.

### 4.9. Description of the Cohort of CRC Primary Tumor Samples Used in This Study

As indicated above, the cohort of 1273 CRC tumor samples from patients including survival data was produced by us in a previous work (see Martinez-Romero et al., 2018) [18]. This is a large integrated cohort of colon primary tumors (*n* = 1273 samples), which included 7 data series of colorectal cancer with genome-wide expression data, plus survival data and other clinical information of the patients. All the colon cancer samples included in this dataset were tested for global gene expression profiling by the platform of high-density microarrays from *Affymetrix*: Human Genome U133 Plus 2.0. As indicated above, the dataset also contained phenotypical and clinical information about the patients, i.e., age at diagnosis, gender, survival time, cancer stage, and tumor location. The samples corresponded in all cases to primary tumors, without pre-operative chemotherapy and/or radiotherapy. Details about the phenotypic data of this CRC cohort are available since they have also been published in Herrera et al., 2021 [41].

### 4.10. Colon Cancer Cohort Risk Prediction and Survival Analysis in Different CMSubtypes

To evaluate the survival and predict the risk for the different groups of CRC tumors classified according to the subtypes (CMSs), a robust version of the multivariate Cox regression model was applied. We used regularized multivariate Cox proportional-hazards regression with L_1_ norm penalty [42], with the scope to build a multigenic risk predictor. A recursive algorithm, using double-nested cross-validation with optimization of regression parameters, searched for the value-of-risk score that best split the cohort into two groups: low risk and high risk. The results of this analysis on CRC tumor samples of subtypes: CMS1 (167 samples) and CMS4 (246 samples) are presented in Figure 5A,B. Once each patient’s risk had been calculated, a Kaplan–Meier analysis checked the separation of the two groups according to the survival data: (i) a high-risk group of individuals (with poor survival, plotted in red) and (ii) a low-risk group of individuals (with good survival, plotted in blue) (presented in Figure 5C,D). A log-rank test evaluated the difference between the Kaplan–Meier curves of the two groups of patients for each gene signature tested. This statistical test is non-parametric and makes no explicit assumptions about the form of the survival curves. A penalty procedure shrunk to zero the coefficient (the *Beta Values*) of any feature of the multivariate model (i.e., any gene) not used to predict the risk. Thus, the multivariate model selected the features (i.e., the genes) that had more power when the prediction was computed, providing a gene risk incidence score for each gene tested in the risk prediction (which can be interpreted as a coefficient of the influence of each gene on the predicted risk) (Figure 5E,F). The genes with highest risk incidence scores (i.e., the genes that showed the largest beta values inside the multivariate model) should split the patients into groups with different prognoses with a statistically significant *p*-value. Individual Kaplan–Meier curves of each gene can be calculated, to test how the expression of each individual gene can divide or split a given cohort of tumor samples into two groups of low-risk and high-risk patients, which correspond to the gene expression level in those samples (as shown in Figure 6). All these procedures and methods were developed and applied using the R (https://www.r-project.org/, last accessed 10 October 2021) programming language for statistical computing.

### 4.11. Quantitative PCR (qPCR) for Gene Expression

RNA isolation, reverse transcription, and qPCR were performed as previously described [39,40]. The amplification of cDNA was achieved by using gene-specific primers (ID2: forward 5′-TCCCACTATTGTCAGCCTGC-3′, reverse 5′-TGAACACCGCTTATTCAGCC-3′; vimentin: forward 5′-CGGGAGAAATTGCAGGAGGA-3′, reverse 5′-AAGGTCAAGACGTGCCAGAG-3′; SNAI2: forward 5′-TGTGTGGACTACCGCTGC-3′, reverse 5′-TCCGGAAAGAGGAGAGAGG-3′; ß2-micro-globulin (B2M): forward 5′-AAAGATGAGTATGCCTGCCGT-3′, reverse 5′-GCTTACATGTCTCG ATCCCA-3′; Homo sapiens glyceraldehyde-3-phosphate dehydrogenase (GAPDH): forward 5′-AAGCTCATTTCCTGGTATGACAACG-3′, reverse 5′-TCTTCCTCTTGTGCTCTTGCTGG-3′) and Quanti Tect SYBR^®^ Green PCR Kits (Qiagen, Hilden, Germany) or Quanti Fast SYBR^®^ Green PCR Kits (Qiagen), according to the manufacturer´s instructions, with a 60 °C annealing temperature. Expression values of ID2 and Vimentin in HCT116 cells were normalized by human B2M, while expression values of SNAI2 in HCT116 cells and ID2, Vimentin and SNAI2 in DLD1 cells were normalized using human GAPDH expression. The gene expressions of p21-/- cells are shown as relative fold expression changes compared to WT controls.

### 4.12. Chorioallantoic Membrane Xenograft Assay

To compare the in vivo growth of HCT116 WT and HCT116 p21-/- cells and evaluate their metastatic potential, a chorioallantoic membrane (CAM) xenograft assay was performed, as recently described by our group [11,39]. Briefly, 1 × 10^6^ cells were engrafted on the CAM of 9-day-old chicken embryos (specific pathogen-free (SPF) eggs; VALO BioMedia, Osterholz-Scharmbeck, Germany) and were allowed to grow into micro-tumors over 5 days. For histological evaluation, the CAM micro-tumors were harvested, fixed in 4% phosphate-buffered formalin, and embedded in paraffin. Thin tissue sections were cut from the formalin-fixed and paraffin-embedded (FFPE) blocks and stained with hematoxylin and eosin, according to the commonly applied standard procedures.

For dissemination, detection cells labeled with a deep-red fluorescent dye (Cell Proliferation Staining Reagent—Deep Red Fluorescence—Cytopainter, Abcam, ab176736) were used and embryos were analyzed using an in vivo optical imaging system (IVIS Spectrum, Perkin Elmer, Waltham, MA, United States) 5 days after engraftment, as previously described by Muenzner et al. [39]. Here, the determined average radiant efficiencies were corrected by the mean auto-fluorescence of chicken embryos engrafted with unlabeled cells, to obtain a measure of metastasis formation.

### 4.13. Plasmids and Transfection

pCEP-WF1 (Addgene plasmid # 16450) and pCEP-WAF1-AS (Addgene plasmid # 16448) were provided by Addgene (Cambridge, MA, USA). Transient transfection was performed as previously described by Lindner et al. (2020) [11], using Lipofectamine^®^ 3000 Transfection Reagent according to the manufacturer’s instruction (Thermo Fisher Scientific, Waltham, MA, USA). Briefly, HCT116 p21-/- cells were seeded to be 70–90% confluent at transfection. Plasmid DNA–lipid complexes were prepared as recommended from the company’s protocol. DNA–lipid complexes were added to the cells and incubated for 48 h. The final plasmid amount was 1.5 µg per well. Hygromycin B treatment was used for transfection selection.

## 5. Conclusions

In this work, for the first time, we show that the mesenchymal HCT116 p21-/- cells exhibit the CMS4 subtype signature of colorectal cancer cells. Moreover, we present evidence that p21-/- cells reflect a rather intermediate EMT phenotype, with an increase in Vimentin and SNAI2 expression, as well as a decrease in E-cadherin expression. The fact that cytoplasmic p21 also contributes to stem cell characteristics and drug resistance shows how carefully p21-dependent effects have to be evaluated and interpreted. We suggest that the gene signature of HCT116 p21-/- cells could be a suitable metric for mechanistic studies regarding the CMS4 signature and its functional consequences in CRC. Our results also indicate that there is a strong correlation between the progression towards the CMS4 phenotype and poor prognosis for colorectal cancer patients.

## Figures and Tables

**Table 1:** Dysregulated genes found by the comparison of HCT116 p21-/- cells versus HCT116 parental WT cells. The top 10 most upregulated genes and top 10 most downregulated genes are presented. All genes and additional information corresponding to the analysis are included in Supplementary Table S1.

Gene Symbol	RefSeq ID	Fold Change	Linear Regression Statistic	Adjusted *p*-Value **	Gene Description
**Upregulated genes**					
*VIM*	NM_003380.2	7.85	1642.29	<10 × 10^−15^	Vimentin; type III intermediate filament protein
*SPARC*	NM_003118.2	8.27	1506.56	<10 × 10^−15^	Secreted prot. acidic and Cys rich; ECM component
*NRP1*	NM_003873.5	3.34	409.97	<10 × 10^−15^	Neuropilin1; receptor prot. involved angiogenesis
*SNAI2*	NM_003068.3	27.30	333.54	<10 × 10^−15^	Snail family transcription repressor 2; zinc finger
*THBS1*	NM_003246.2	2.23	281.49	<10 × 10^−15^	Thrombospondin; cell-cell & cell-matrix interaction
*TNC*	NM_002160.3	32.20	251.40	<10 × 10^−15^	Tenascin C
*PLEKHO1*	NM_016274.4	1.48	140.07	<10 × 10^−15^	Pleckstrin homology domain containing O1
*FGF2*	NM_002006.4	1.59	124.22	<10 × 10^−15^	Fibroblast growth factor 2
*FERMT2*	NM_001135000.1	1.33	113.17	<10 × 10^−15^	Fermitin family member 2
*SPHK2*	NM_020126.3	1.73	103.01	<10 × 10^−15^	Sphingosine kinase 2
**Downregulated genes**					
*ID2*	NM_002166.4	−4.65	972.57	<10 × 10^−15^	Inhibitor DNA binding 2; negat. reg. bHLH TFs
*TP53*	NM_000546.2	−4.06	804.76	<10 × 10^−15^	Tumor protein 53; tumor suppressor
*IL18*	NM_001562.2	−4.79	734.54	<10 × 10^−15^	Interleukin 18; pro-inflammatory cytokine
*RAMP1*	NM_005855.2	−32.20	667.65	<10 × 10^−15^	Receptor activ. modif. protein 1; calcitonin GPCR
*CDH1*	NM_004360.2	−3.39	617.59	<10 × 10^−15^	Cadherin 1; cell surface adhesion molecule
CDKN1A *	NM_000389.2	−3.60	552.82	<10 × 10^−15^	Cyclin-dependent kinase inhibitor 1A (*p21 KO)
*ITGA3*	NM_002204.2	−3.09	352.35	<10 × 10^−15^	Integrin subunit alpha 3
*TJP3*	NM_014428.1	−3.25	350.49	<10 × 10^−15^	Tight junction protein 3
*S100A14*	NM_020672.1	−3.22	294.47	<10 × 10^−15^	S100 calcium binding protein A14
*GRHL2*	NM_024915.3	−3.89	291.45	<10 × 10^−15^	Grainyhead-like transcription factor 2

Official Full Names (from NCBI GENE): *VIM*: vimentin; *SPARC*: secreted protein acidic and cysteine rich; *NRP1*: neuropilin 1, receptor protein involved in angiogenesis; *SNAI2*: snail family transcriptional repressor 2; *THBS1*: thrombospondin 1; *TNC*: tenascin C; *PLEKHO1*: pleckstrin homology domain containing O1; *FGF2*: fibroblast growth factor 2; *FERMT2*: fermitin family member 2; *SPHK2*: sphingosine kinase 2; *ID2*: inhibitor of DNA binding 2; *TP53*: tumor suppressor protein 53; *IL18*: interleukin 18; *RAMP1*: receptor activity modifying protein 1; *CDH1*: cadherin 1; *CDKN1A*: cyclin-dependent kinase inhibitor 1A (is the gene knockout, KO, of this study, that encodes the *p21 protein); *ITGA3*: integrin subunit alpha 3; *TJP3*: tight junction protein 3; S100A14: S100 calcium binding protein A14; *GRHL2*: grainyhead-like transcription factor 2. ** Note that all the adjusted *p*-values were highly significant, resulting all below the 10 × 10^−15^ threshold.

**Table 2:** Functional enrichment analysis of selected genes with concurrent annotations.

General Function	Genes	Query Lists	Reference Lists	Enrichment *p*-Value	Silhouette Similarity	Functional Terms Assigned (Concurrent Enrichment)
**Upregulated genes**						
**– Fibroblast** **phenotype,** **cytoskeleton, cancer**	*PIK3CA, PLCG1, FGF18, FGF2, PDGFA*	5 (23)	50 (34,208)	1.79 × 10^−10^	0.7500	– **GO:0008543:** fibroblast growth factor receptor signaling pathway (BP); **Kegg: 04810:** regulation of actin cytoskeleton; **Kegg: 05218:** melanoma.
**– ECM changes**	*THBS1, SPARC, LAMC1, TNC, OLFML2B, MMP13*	6 (23)	134 (34,208)	3.08 × 10^−10^	0.9129	– **GO:0031012:** extracellular matrix (CC); **GO:0005576**: extracellular region (CC).
**– Cell adhesion**	*THBS1, TNC, LAMC1, PDGFA, PIK3CA*	5 (23)	197 (34,208)	1.86 × 10^−7^	0.8944	– **Kegg:04510:** focal adhesion.
**Downregulated genes**						
**– Cell contact** **regulation**	*CD44, ITGA2, ITGA3, ITGB4, ADAM15* *PTK2B, RAMP1, DSC2*	8 (40)	93 (34,208)	1.57 × 10^−13^	0.7335	– **GO:0009986:** cell surface (CC); **GO:0007160:** cell-matrix adhesion (BP); **Kegg: 04512:** ECM and receptor interaction; **GO:0007229:** integrin-mediated signaling pathway (BP); **Kegg: 05412:** arrhythmogenic right ventricular cardiomyopathy.
**– Cancer genes**	*CDKN1A(p21), TP53, CDH1, MET, PDGFC, TYMP*	6 (40)	63 (34,208)	1.12 × 10^−10^	0.7717	– **Kegg: 05218:** melanoma; **Kegg: 05219:** bladder cancer.
**– Cell adhesion** **and cytoskeleton**	*ITGA2, ITGA3, ITGB4, MET, PDGFC*	5 (40)	111 (34,208)	1.97 × 10^−7^	0.7500	– **Kegg: 04510:** focal adhesion; **Kegg: 04810:** regulation of actin cytoskeleton.

## Data Availability

The expression data for HCT116 cells are available at GEO (Gene Expression Omnibus database, https://www.ncbi.nlm.nih.gov/geo/ last accessed 22 December 2021) with via the reference number GSE135923.

## Supplementary Materials

The following supporting information can be downloaded at: https://www.mdpi.com/article/10.3390/cancers14010136/s1. Supp. Figure S1: Representative images of chicken embryos as assessed using an in vivo fluorescence imaging system 5 days after engraftment with fluorescently labeled HCT116 WT and HCT116 p21-/- cells. Corrected average radiant efficiency determined through fluorescence imaging as a measure for the in vivo metastatic potential of HCT116 WT (*n* = 9) and HCT116 p21-/- (*n* = 10) cells. Supp. Figure S2: Kaplan–Meier survival analysis of *ITGB4* identified in the risk prediction of CRC tumor samples of CMS1 subtype (167 samples). (A,B) Kaplan–Meier (KM) curve corresponding to the samples divided by the expression level of the gene (high expression red, low expression green) with (A) CMS1 and (B) CMS4 tumors. Supp. Table S1: Complete list of 67 deregulated genes in HCT116 p21-/- cells, including differential expression statistical parameters, the assignment of each gene to CRC subtypes CMS1 and CMS4, and gene IDs and description. Supp. Table S2: Gene sets used in the functional enrichment analysis performed to identify the biological processes that were overrepresented in the subsets of CRC samples assigned to CMS1 and CMS4. The uncropped western blot figures can been found in supplementary files. File S1: Uncropped Western Blot Figures.
