# Supplementary material for: A Gene Signature Derived from the Loss of CDKN1A (p21) Is Associated with CMS4 Colorectal Cancer
Source: Cancers (Basel). 2021 Dec 28;14(1):136. doi: 10.3390/cancers14010136 (PMC8750372; doi:10.3390/cancers14010136)
Supplement: Supplementary file 1 [file cancers-14-00136-s001.zip › cancers-1497702/Supplementary Files/File S1-Uncropped Western Blot Figures.pdf]

Figure 1C HCT116

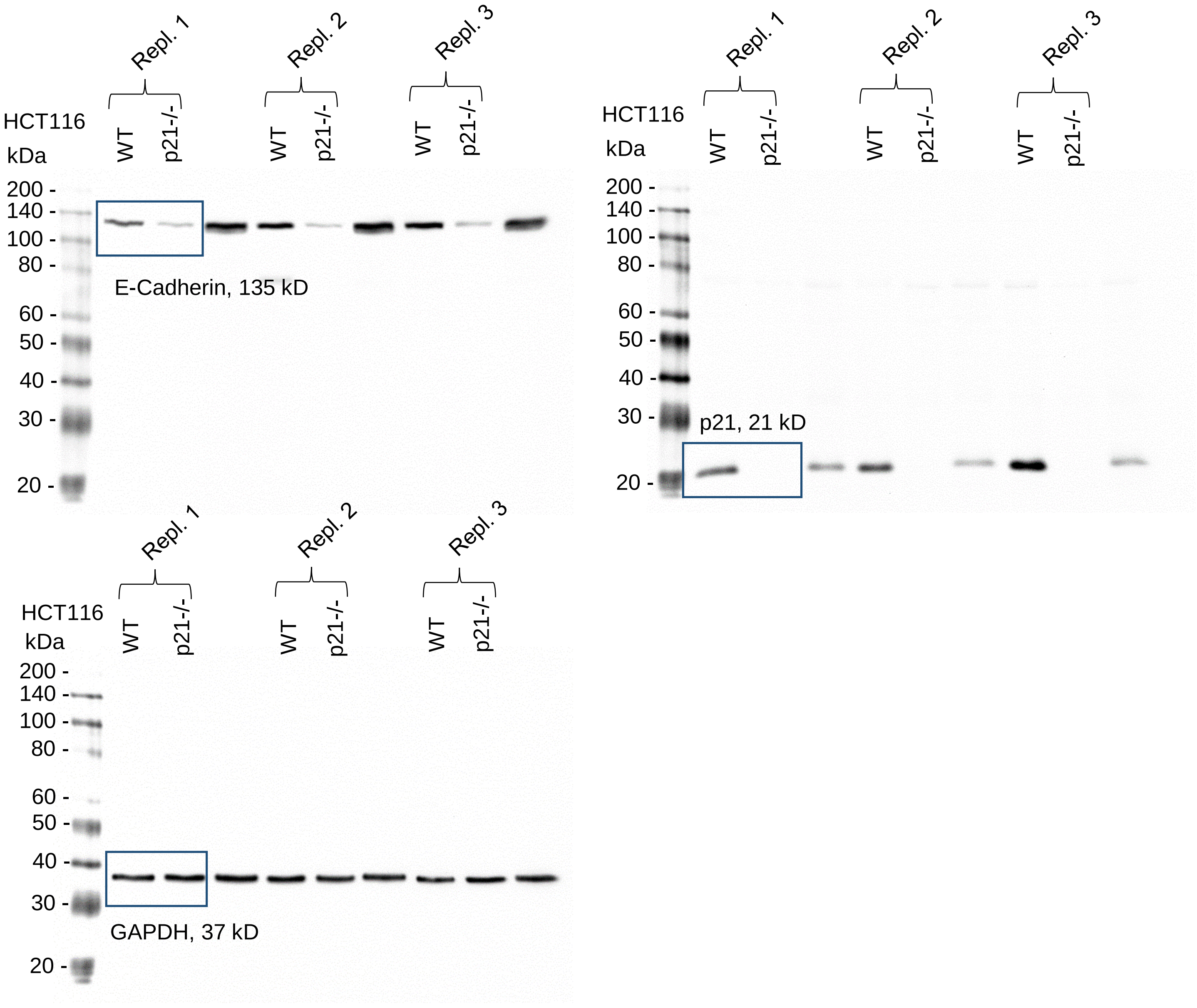

Figure 1C HCT116 (cont.)

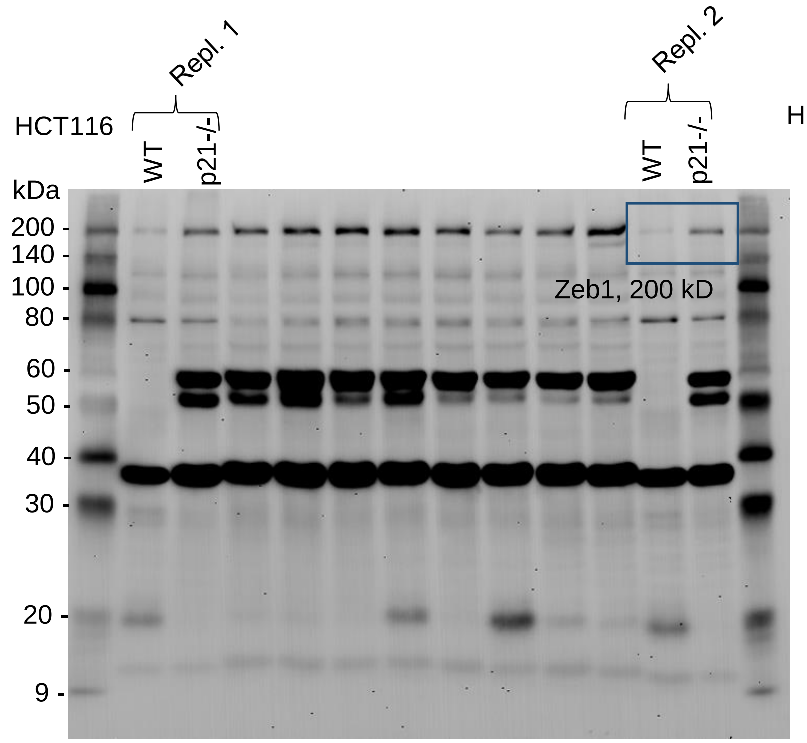

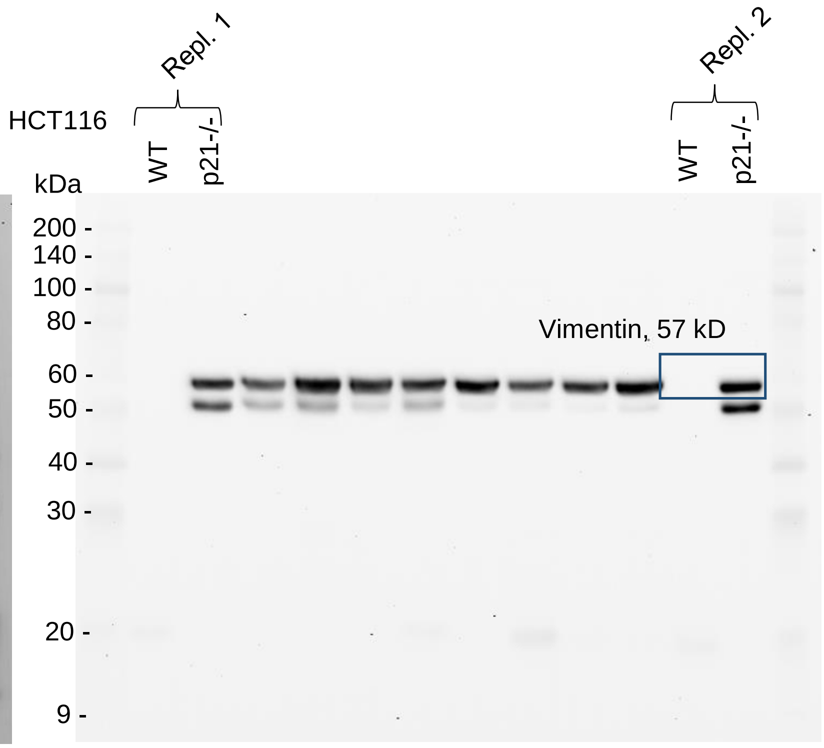

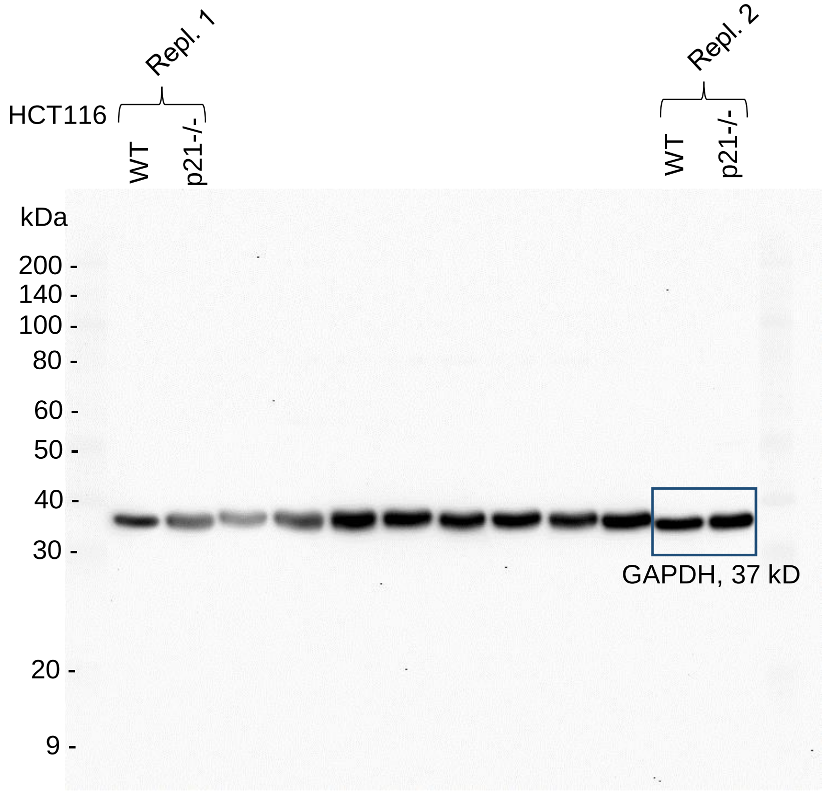

Figure 1D DLD1

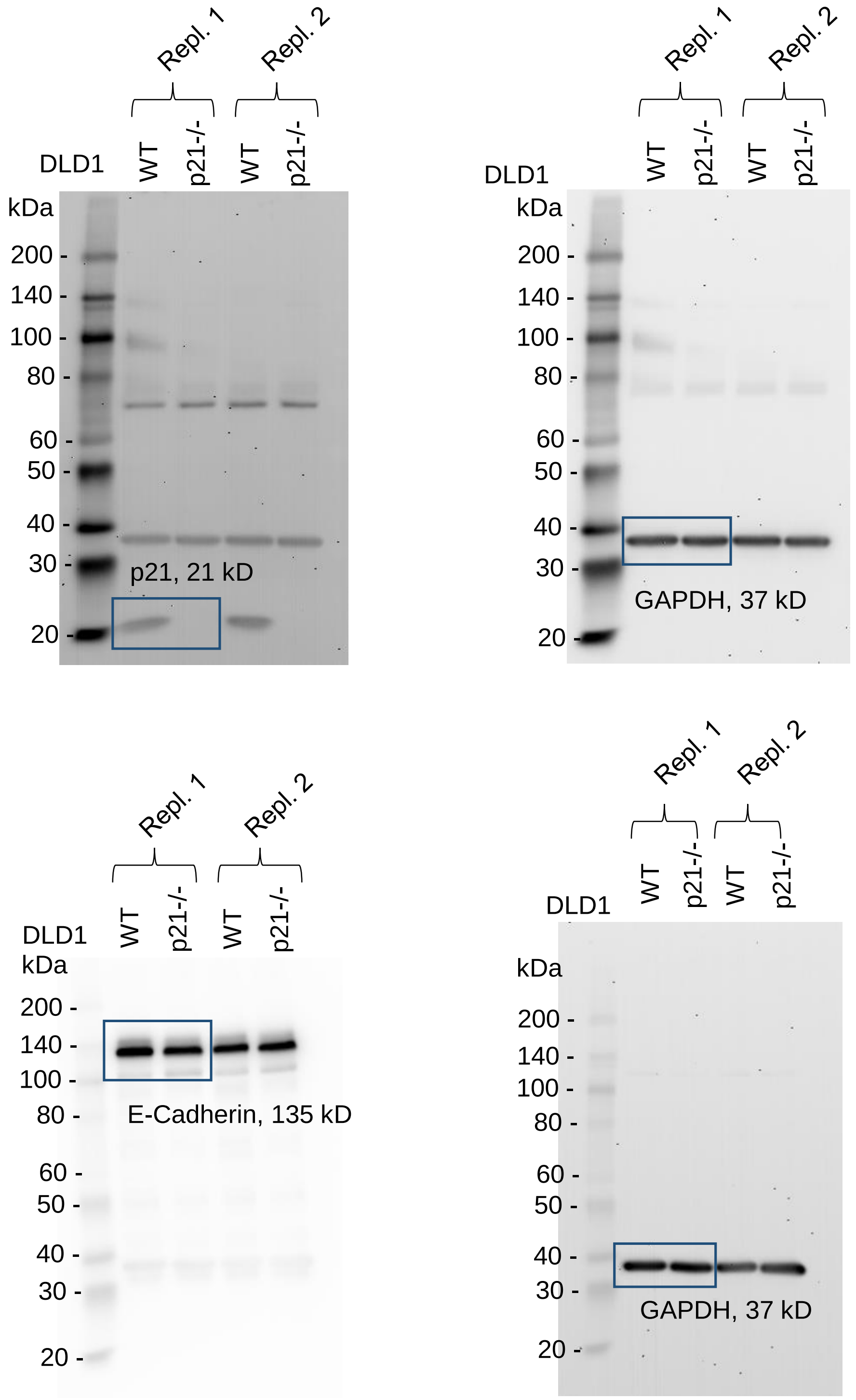

Figure 1F HCT116

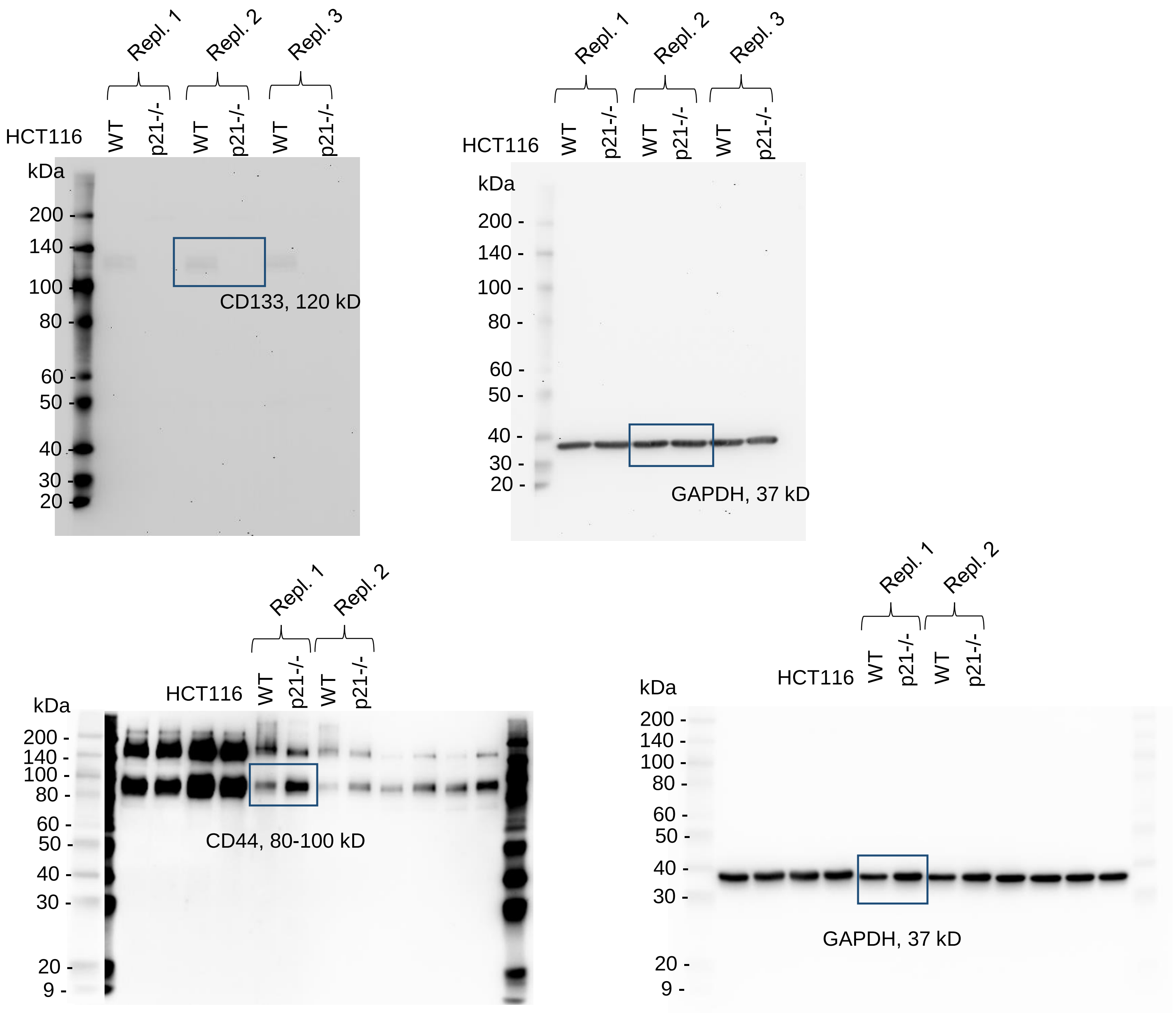

Figure 1F HCT116 (cont.)

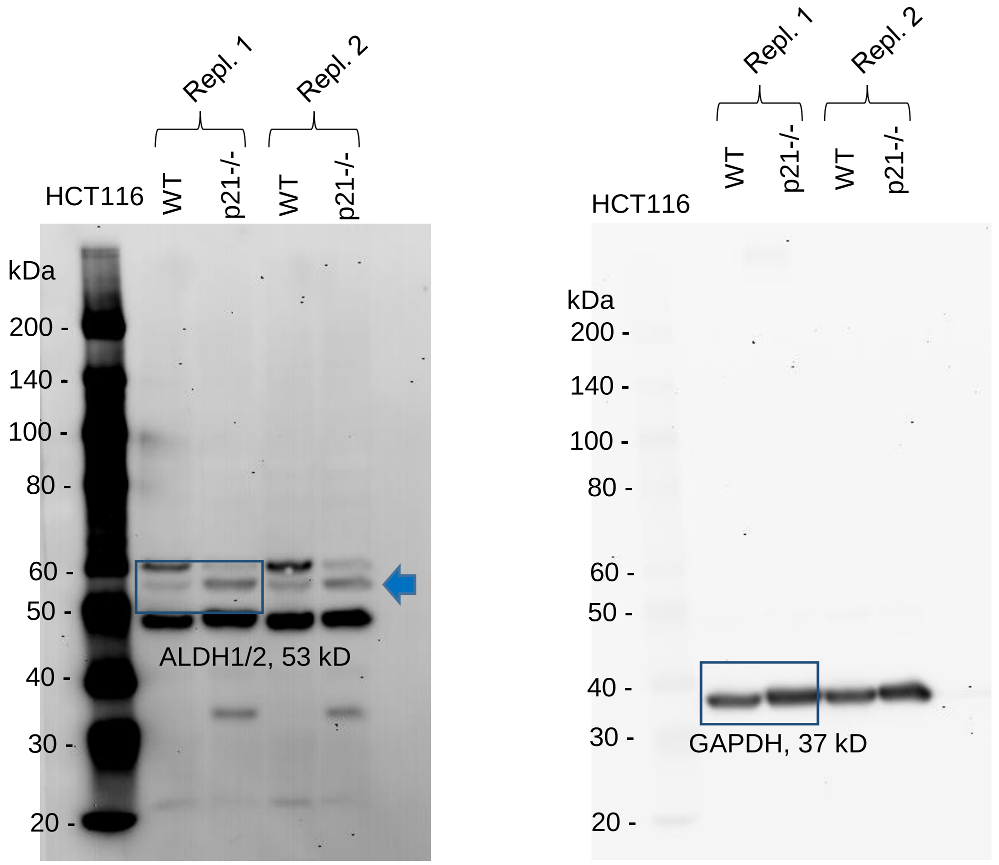

Figure 1F DLD1

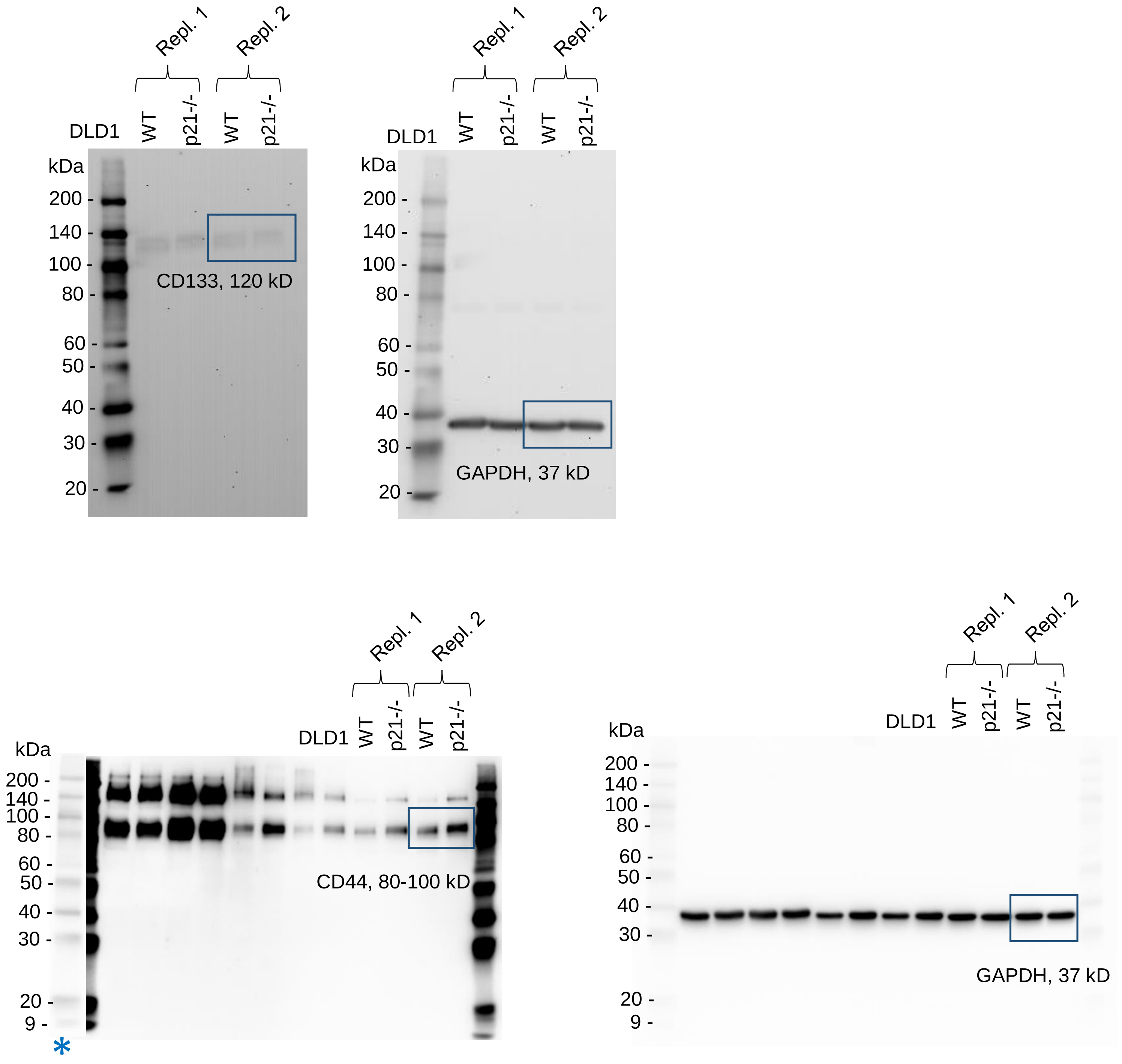

* The ladder from earlier or later exposure time was use to better indicate the molecular weight of proteins.

Figure 1F DLD1 (cont.)

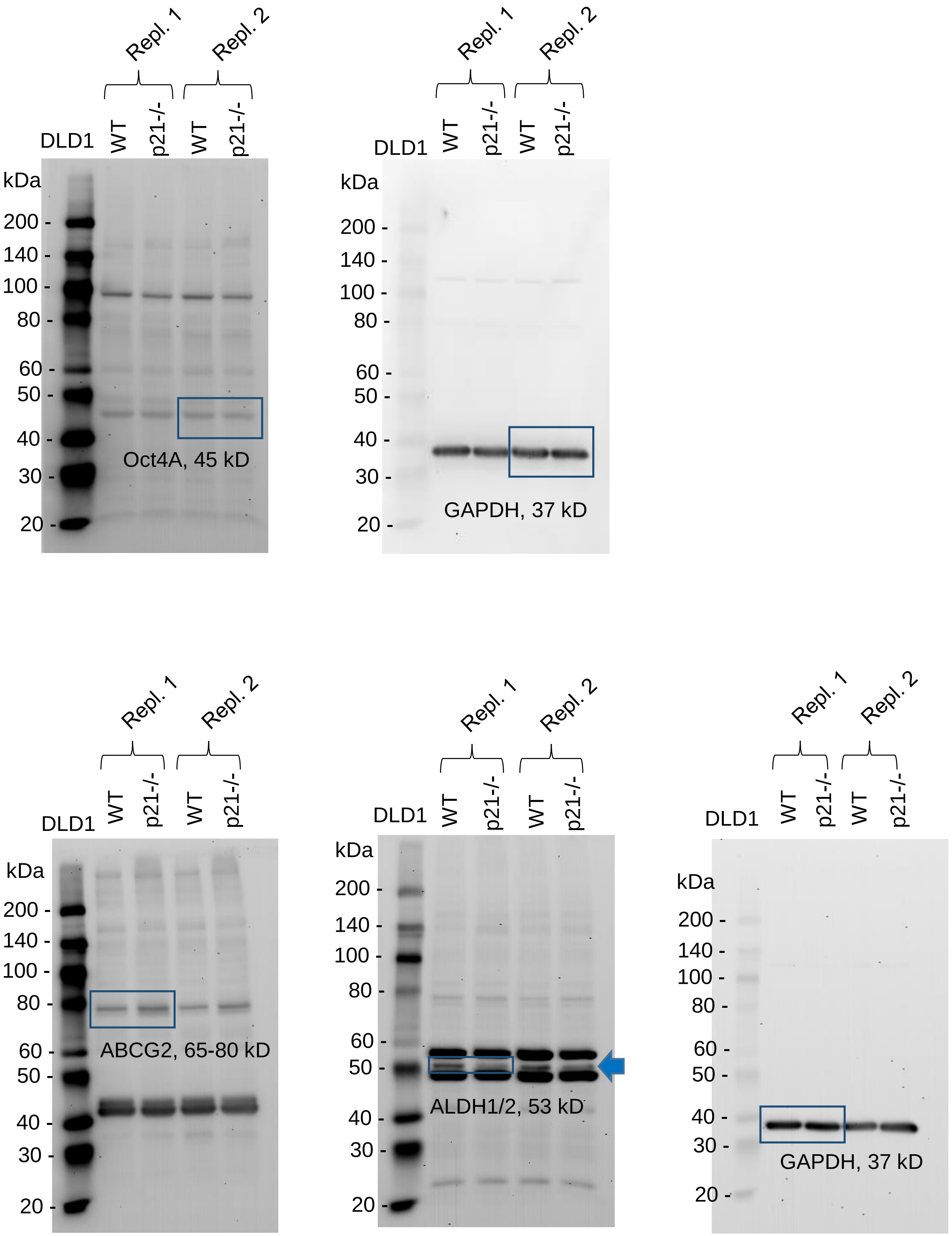

Figure 2D HCT116

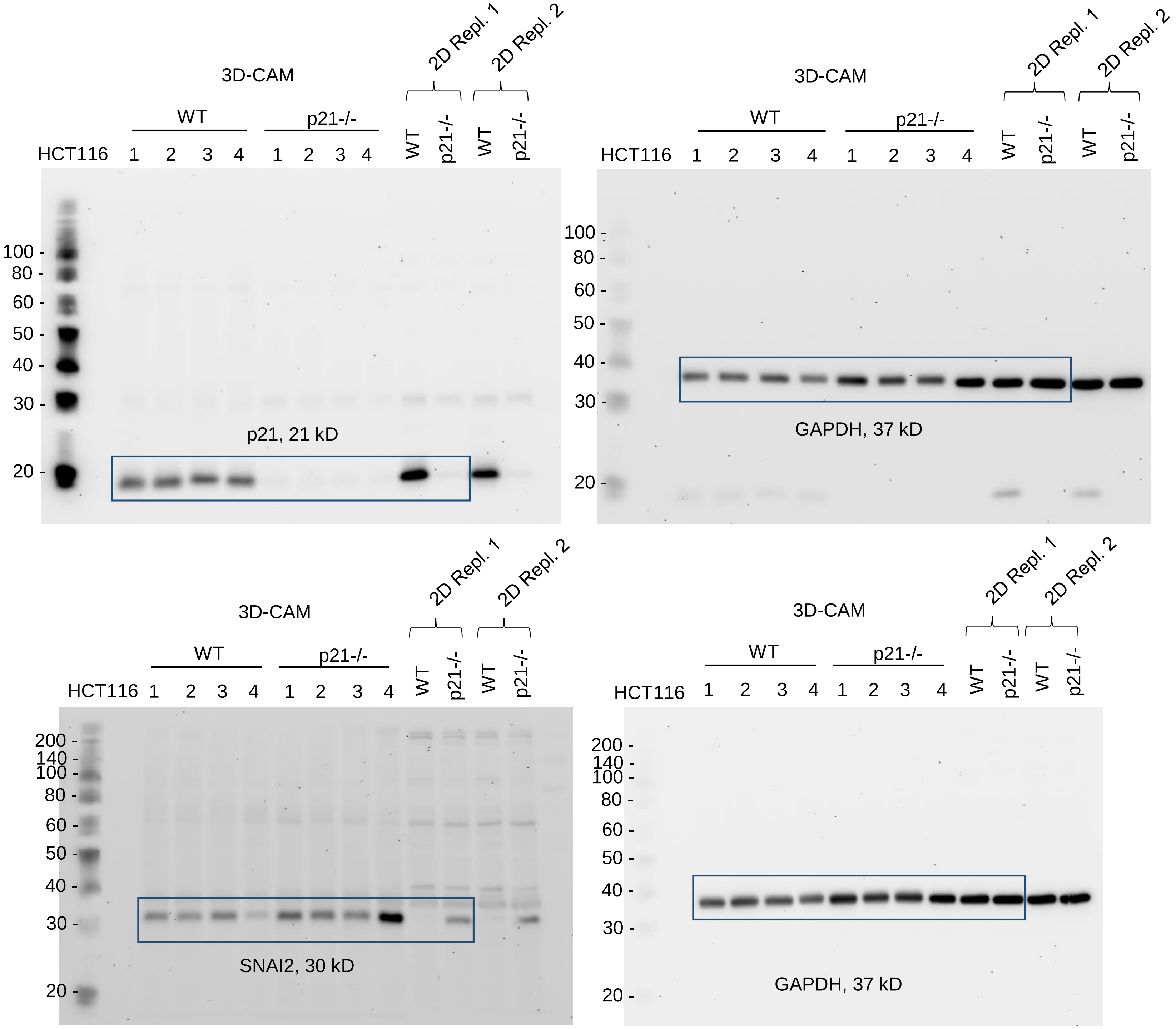

Figure 2E p21 over-expression HCT116 p21-/-

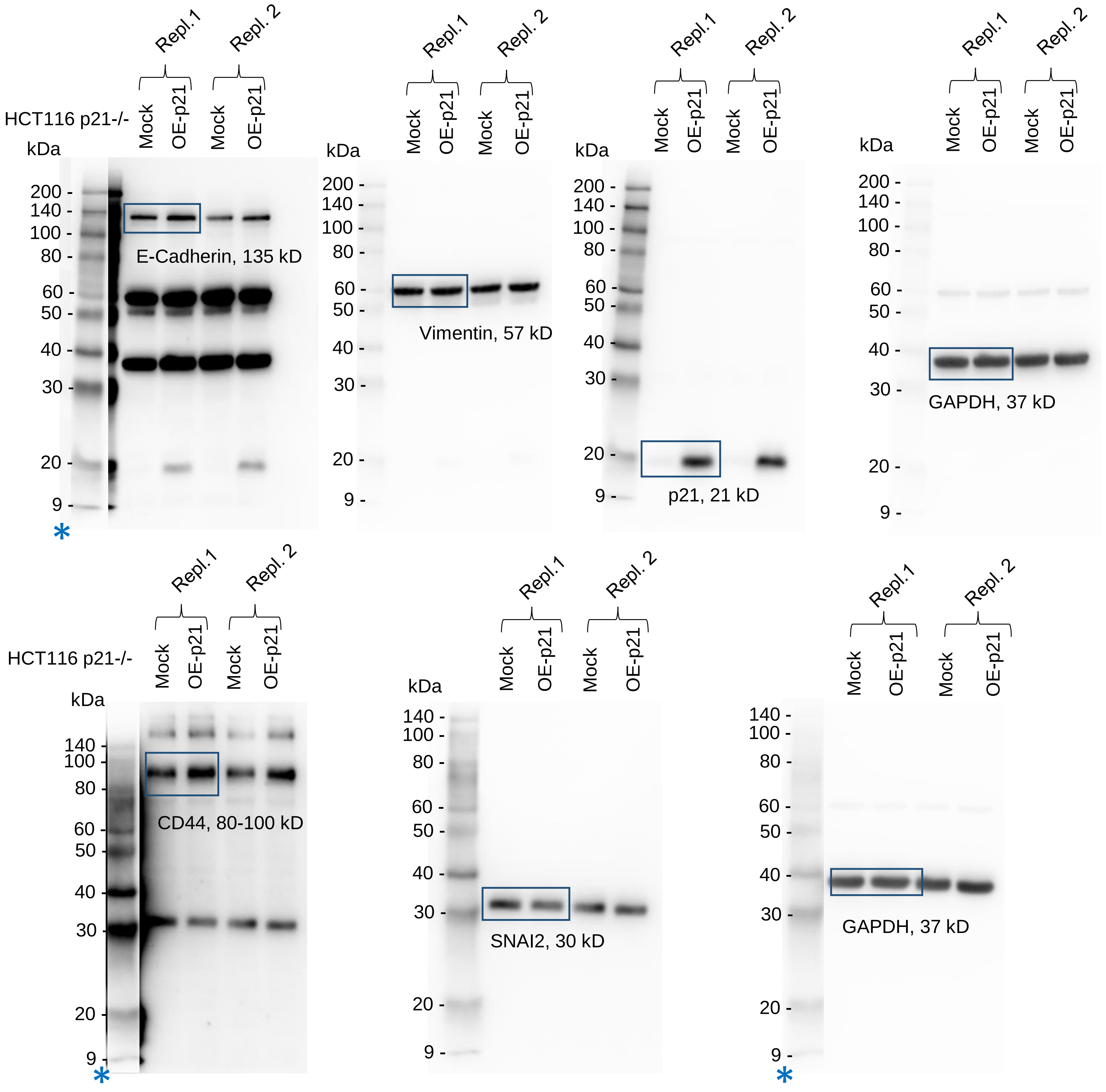

* The ladder from earlier or later exposure time was use to better indicate the molecular weight of proteins.

Figure 2E p21 over-expression HCT116 p21-/- (cont.)

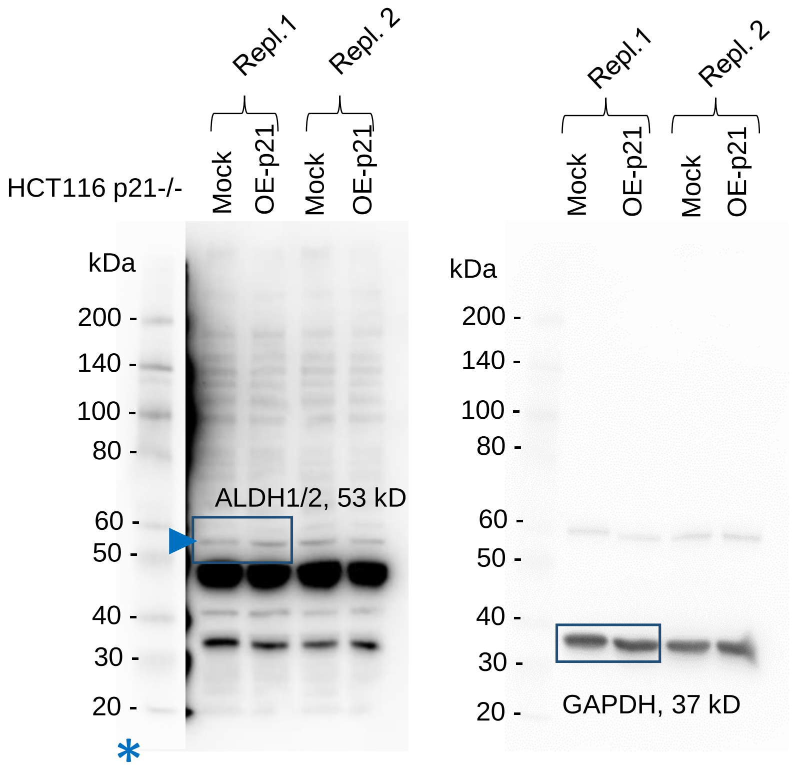

* The ladder from earlier or later exposure time was use to better indicate the molecular weight of proteins.
