# Supplementary material for: A Gene Signature Derived from the Loss of CDKN1A (p21) Is Associated with CMS4 Colorectal Cancer
Source: Cancers (Basel). 2021 Dec 28;14(1):136. doi: 10.3390/cancers14010136 (PMC8750372; doi:10.3390/cancers14010136)
Supplement: Supplementary file 1 [file cancers-14-00136-s001.zip › cancers-1497702/Supplementary Files/Supplementary-Figure-S2.pdf]

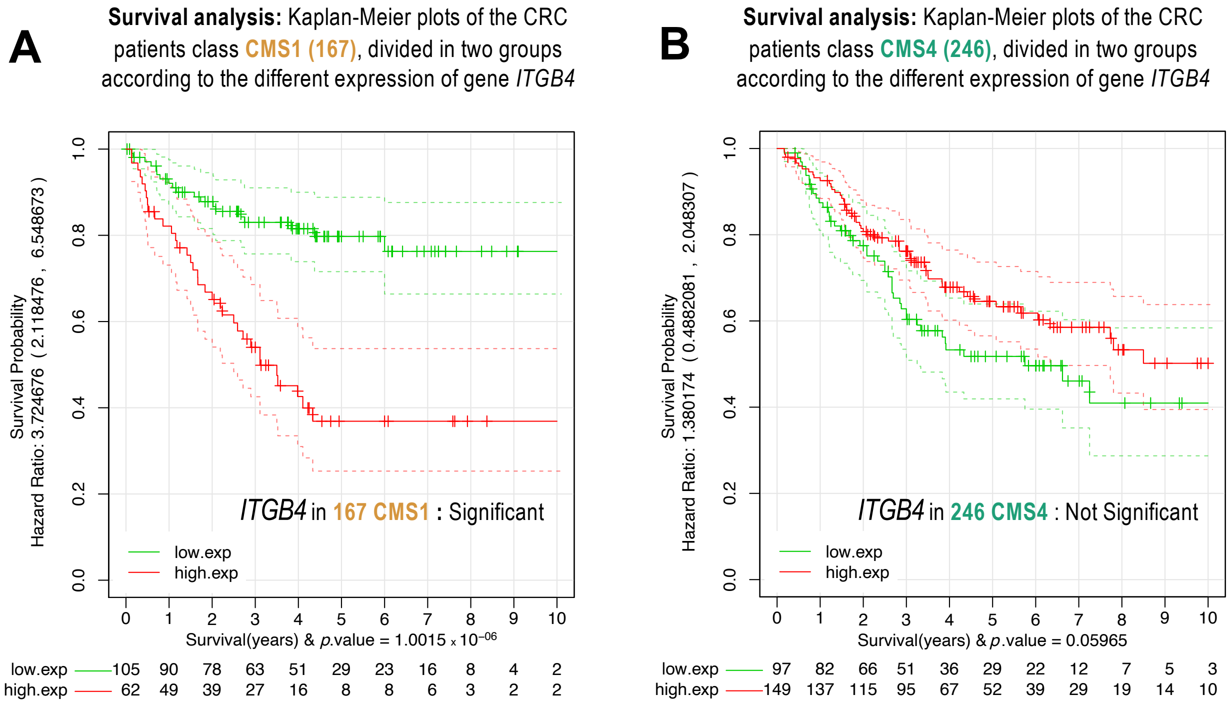
A
Survival analysis: Kaplan-Meier plots of the CRC patients class CMS1 (167), divided in two groups according to the different expression of gene ITGB4
Survival Probability
Hazard Ratio: 3.724676 (2.118476 , 6.548673)
ITGB4 in 167 CMS1 : Significant
low.exp
high.exp
Survival(years) & p.value = 1.0015 x 10−06
low.exp 105 90 78 63 51 29 23 16 8 4 2
high.exp 62 49 39 27 16 8 8 6 3 2 2
B
Survival analysis: Kaplan-Meier plots of the CRC patients class CMS4 (246), divided in two groups according to the different expression of gene ITGB4
Survival Probability
Hazard Ratio: 1.380174 (0.4882081 , 2.048307)
ITGB4 in 246 CMS4 : Not Significant
low.exp
high.exp
Survival(years) & p.value = 0.05965
low.exp 97 82 66 51 36 29 22 12 7 5 3
high.exp 149 137 115 95 67 52 39 29 19 14 10
