# Supplementary figures and images for: A Gene Signature Derived from the Loss of CDKN1A (p21) Is Associated with CMS4 Colorectal Cancer
Source: Cancers (Basel). 2021 Dec 28;14(1):136. doi: 10.3390/cancers14010136 (PMC8750372; doi:10.3390/cancers14010136)

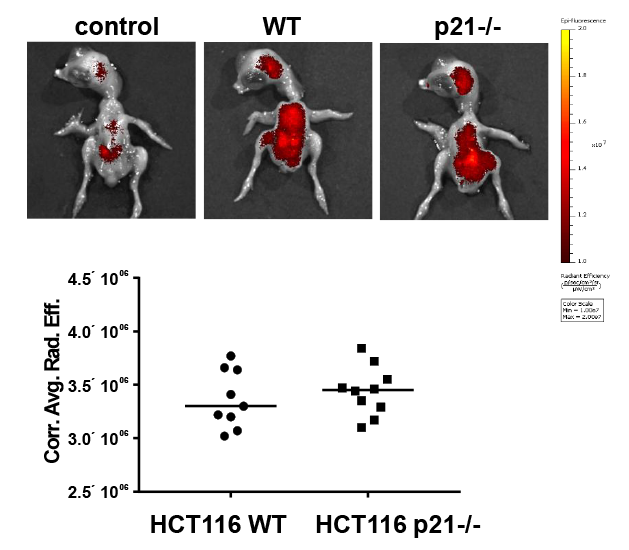

Supplement: Supplementary file 1 [file cancers-14-00136-s001.zip › cancers-1497702/Supplementary Files/Supplementary-Figure-S1.tif]
